# Rivers under stress: geochemical changes along a rural–urban gradient during extreme hydrological events (Greve River, Italy)

**DOI:** 10.1007/s10653-026-03283-9

**Published:** 2026-06-17

**Authors:** M. Ferrari, R. Biagi, S. Venturi, F. Frezzi, F. Tassi

**Affiliations:** 1 Department of Earth Sciences, University of Florence, Via G. La Pira 4, 50121 Florence, Italy; 2 Istituto di Geoscienze e Georisorse (IGG), Consiglio Nazionale delle Ricerche (CNR), Florence, Italy

**Keywords:** Drought-flood transition, First flush processes, Contaminant remobilization, Anthropogenic contamination, Trace elements, Stable isotopes

## Abstract

Rivers are increasingly exposed to combined anthropogenic and climate-driven pressures, particularly along rural-to-urban gradients where land use, population density, and contaminant sources progressively intensify downstream. In parallel, prolonged droughts followed by intense rainfall events are becoming more frequent, potentially enhancing contaminant accumulation during dry periods and their rapid mobilization during subsequent first-flush events. However, the interactions between these hydrological extremes and spatial anthropogenic gradients in controlling river geochemistry remain insufficiently understood. This study investigates the spatial and seasonal evolution of river chemistry along the Greve River (Tuscany, Italy), a tributary of the Arno River, characterized by a marked rural-to-urban transition, during the exceptional 2022 European drought and subsequent heavy rainfall event. Six sampling campaigns were conducted between May and October 2022, combining high-resolution spatial monitoring with geochemical and isotopic tracers, including total dissolved solids (TDS), nutrients, and trace elements. The results reveal a progressive downstream enrichment in anthropogenic tracers (e.g., NO_3_^−^ and K^+^, up to 9 mg/L and 6.6 mg/L, respectively) and pronounced evaporative isotope enrichment during the driest months (slope of the evaporation line ~ 4), indicating strong hydrological stress conditions. The first intense rainfall event triggered rapid remobilization of accumulated material and heterogeneous contaminant transport dynamics. Elevated nutrient concentrations (e.g., NH_4_^+^, up to 1.4 mg/L) in downstream sectors further reflect the combined influence of hydrological stress and urban inputs. These findings demonstrate how climate-driven hydrological extremes can amplify contaminant mobilization along urbanized river continuums. The integration of isotopic and geochemical tracers provides a transferable framework for assessing water-quality vulnerability in river basins increasingly exposed to hydroclimatic instability.

**Supplementary Information:**

The online version contains supplementary material available at 10.1007/s10653-026-03283-9.

## Introduction

Rivers have always been at the heart of human settlements, providing essential resources for domestic uses (~ 10%), and sustaining agricultural (~ 70%) and industrial (~ 20%) activities (UNESCO, 2024). Historically, major urban centers worldwide have preferentially developed along rivers, with the organization and distribution of settlements closely linked to the morphology of the river basin (Ridolfi et al., 2025). This structure typically generates a pronounced downstream gradient in land use and anthropogenic pressure, ranging from predominantly natural and vegetated headwaters to densely populated and industrialized lowland areas, which act as major sources of water pollution (Zhang et al., 2025; Zingraff-Hamed et al., 2021). In parallel, climate change is altering hydrological regimes by increasing the frequency and intensity of extreme events, including prolonged droughts and intense precipitation episodes, while also enhancing rainfall variability and unpredictability (Ponnuchakkammal et al., 2026). Prolonged dry periods increase water residence time and promote the accumulation of solutes and particulate-bound contaminants within catchments, whereas subsequent high-intensity rainfall events can rapidly mobilize and export these accumulated materials downstream (Müller et al., 2021).

Despite extensive research on riverine pollution along rural–urban gradients (Byekwaso et al., 2026; Hariwati et al., 2026; Horve et al., 2026), the combined influence of prolonged drought conditions and subsequent intense rainfall events on the spatial redistribution and transport of dissolved and particulate-associated contaminants along entire river continuums remains poorly constrained. This study addresses this gap by investigating the Greve River, a major tributary of the Arno River (Tuscany, Italy), characterized by a well-defined upstream-to-downstream transition from rural to highly urbanized conditions. This setting represents a transferable model system for many temperate river basins subjected to similar land-use gradients and climate forcing. The study combines high-resolution spatial sampling along the river with geochemical and isotopic analyses conducted under contrasting hydrological conditions, including an exceptionally prolonged drought followed by an intense rainfall event, which occurred during summer 2022 across Italy and much of Europe.

Six sampling campaigns were conducted between May and October 2022, enabling the characterization of both spatial variability and hydrological phase-dependent geochemical responses from low- to high-flow conditions. By integrating isotopic signatures with multi-element geochemical tracers across contrasting hydrological phases, this study provides new insights into the relative roles of sediment remobilization, dissolved transport, and spatially heterogeneous anthropogenic inputs under climate-driven hydrological extremes.

## Study area

**Fig. 1:**
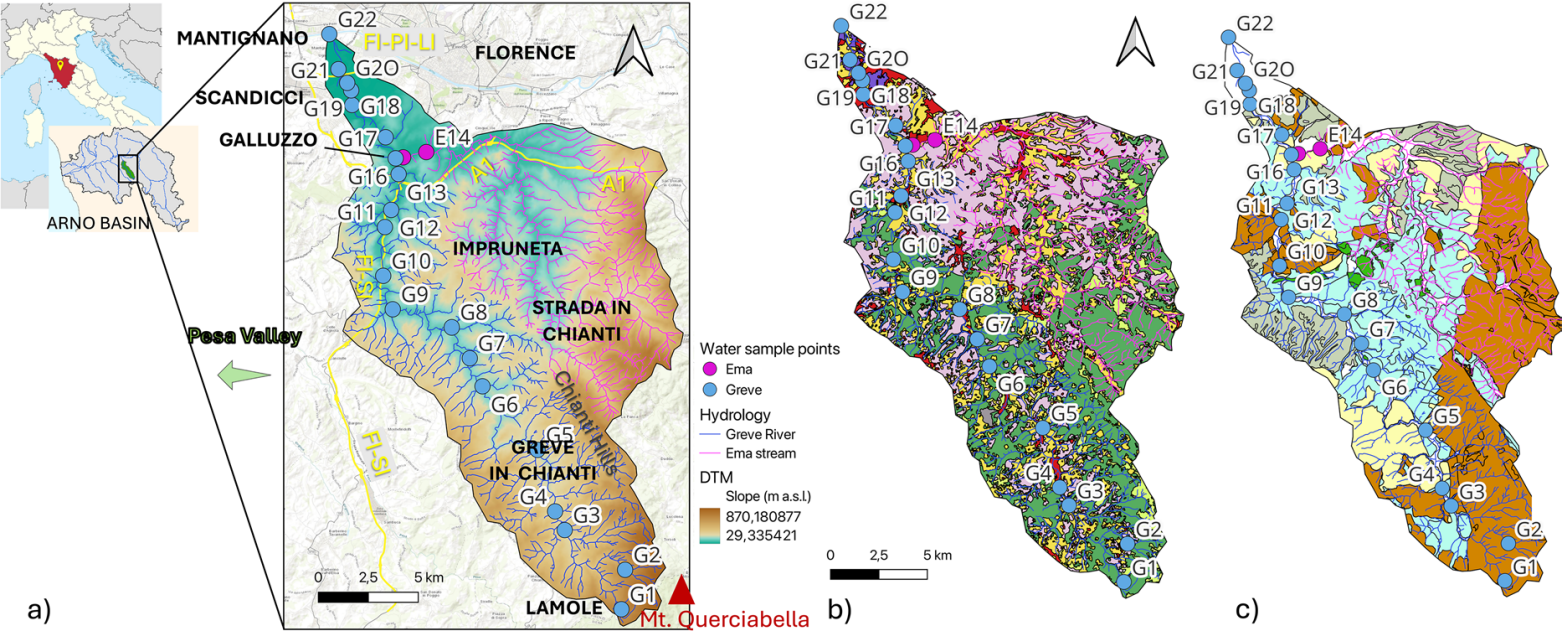
**a** Map of the Greve River Basin showing water sampling points, control points (orange markers), and rainwater collectors (yellow stars). **b** Land use map including forests (dark green), croplands (yellow), grasslands (light green), olive groves and vineyards (pink), mining areas (grey), plant nurseries and greenhouses (purple), and urban areas (red). **c** Geological map of the study area showing sandstones (brown), carbonatic flysch and marly limestones (light yellow), mudstones and calcilutites (light blue), basalts, peridotites and ophiolitic breccias (green), and unconsolidated sands and gravels (grey)

The Greve River Basin (GRB, Fig. 1a), a left tributary of the Arno River (Tuscany, Italy), has an area of 283 km^2^ and encompasses a tributary, the Ema stream, whose basin is 121 km^2^. The total length of the Greve River, which has undergone significant transformation over the years due to progressive urbanization and infrastructure development in the territory, is approximately 50 km. It originates from Mount Querciabella, at an elevation of 845 m a.s.l. (above sea level). In the first stretch, it flows north-westward with a torrential regime on steep slopes dominated by woods, where minor villages and localities devoted to wine production and enological tourism, e.g., the locality of Lamole at c.a. 600 m a.s.l., occur (Fig. 1a and b). Reaching the village of Greve in Chianti, the river enters a wide valley flowing through the Chianti Hills Territory (ca. 300 m a.s.l.), a rural area hosting about 28,000 inhabitants primarily devoted to agriculture, with a prevalence of olive trees and vineyard cultivation alternated with small woods covering the steepest areas (Fig. 1b). Clay excavation and handicraft activities, keen on the production of a ceramic material known as Cotto Imprunetino, occur at Impruneta village (ca. 130 m a.s.l.), whereas downstream, at ca. 100 to 75 m a.s.l., the basin is bordered to the west by the watershed separating the GRB from the Pesa stream basin and flows north-eastward close to the highway connecting Florence to Siena (FI-SI highway; Fig. 1a). The Greve River receives waters of the Ema stream on its right bank at the Charterhouse of Florence, located close to the residential village of Galluzzo (ca. 58 m a.s.l.), which is a hub for urban traffic connecting the city center with the A1 Milan-Naples highway and the southern territories. The proper urban stretch of the river begins at Scandicci town (ca. 50 m a.s.l.) and, about 4 km downstream, joins the Arno River on its left bank in the locality of Mantignano, Florence (Fig. 1a).

The Greve basin is a wide tectonic depression filled by fluvio-lacustrine deposits up to 550 m thick. The eastern part of the basin is characterized by a relatively high relief energy, dictated by the geology of the substrate, which mainly consists of sandstone, silts, calcilutites, and marls of the Tuscany Nappe formations (Macigno and Scaglia Toscana) (Fig. 1c). In the central part of the basin, rock formations belonging to the Ligurian domain, consisting mainly of mudstones, calcilutites, carbonate flysch, and marls outcrops (Fig. 1c). Near the Impruneta village, outcrops from both the Tuscan Nappe and the Ligurian formation are recognized, the latter comprising serpentinites, gabbros, and peridotites (Fig. 1c).

The flat sector of the basin, corresponding to the urban area and its suburban surroundings, experiences a temperate climate, characterized by mild winters and hot, humid summers, during which temperatures may exceed 40 °C. In contrast, the hilly sector toward the river source is characterized by relatively cold winters, with temperatures occasionally dropping below 0 °C, and dry, scorching summers, when temperatures can rise above 35 °C (Venturi et al., 2020). Annual rainfall in the plain and the hilly areas is 850–900 and 950–1000 mm, respectively (Diodato et al., 2021).

## Sampling strategy, materials, and analytical methods

Six sampling campaigns were carried out in 2022, as follows: (A) May 24, (B) June 30, (C) July 07, (D) August 22, (E) September 28, and (F) October 24. The first and the last campaign (A and F) included 20 sampling points from the Greve river (from G1 to G22, from source to mouth), and 2 from the Ema stream (E14 and E15; Fig. 1a), whereas during the other campaigns samples were collected from five points (G4, G11, E15, G18, G22; Fig. 1a), selected based on the analytical results obtained from the first monitoring campaign.

The first two months of the sampling period (May and June) were marked by an extreme drought, followed in July by a local rainfall event that affected just the Ema sub-basin. In August, the sampling took place after an intense rain event that impacted the entire GRB, whereas September and October sampling campaigns were carried out after numerous rainfalls of low-to medium intensity (see Figure S1 for the precipitation time series plots).

Electrical conductivity, Oxidation–Reduction Potential (ORP), and pH of river water samples were measured in situ using a Revio© multiprobe (XS Sensor). For laboratory analyses, different water aliquots were collected, as follows: (i) one sample in a 100 mL HPDE bottle for major anions analysis; (ii) one acidified (with 0.5 mL of 30% Suprapure HCl) and filtered (0.45 µm) sample in a 50 mL HPDE bottle for major cations analysis, (iii) one acidified (with 0.5 mL of 65% Ultrapure HNO_3_) and filtered (0.45 µm) sample in a 50 mL HPDE bottle for the analysis of trace elements; (iv) one acidified (with 0.5 mL of 95% Suprapure H_2_SO_4_) and filtered (0.45 µm) sample in a 50 mL HPDE bottle for chemical oxygen demand (COD) and total dissolved phosphorus (P_tot_) determination; (v) one filtered (0.45 µm) sample in a 15 mL HPDE vial carefully filled to avoid air bubbles for the analyses of δ^2^H and δ^18^O values; (vi) one sample in a 10–12 L plastic canister for the determination of the suspended load (TSS). Aliquots (iv) and (vi) were collected only at the five selected points (yellow stars in Fig. 1a) during all six samplings.

Main anions (F^−^, Cl^−^, Br^−^, NO_3_^−^, and SO_4_^2−^) and cations (Ca^2+^, Mg^2+^, Na^+^, and K^+^) were analyzed by ion chromatography (IC), using Metrohm 761 and Metrohm 861 chromatographs, respectively, while alkalinity was measured by acidic titration (AT) using HCl 0.01 M and methyl orange as indicator (Metrohm Titrino). Nitrites (Nitriver3 Method) and ammonium (Nessler Method) were analyzed by molecular spectrophotometry (MSP; HACH DR/2010). COD (Reactor Digestion Method 8000; Jirka et al., 1975), and P_tot_ (Test’N Tube Method 8190) were analyzed by MSP. The analytical errors for AT, IC, and MSP were < 5%. Trace elements were measured by Inductively Coupled Plasma Mass Spectrometry (ICP-MS) using an Agilent 7800 Mass Spectrometer with an analytical error below 10%. The isotopic ratios ^18^O/^16^O and ^2^H/^1^H (expressed as δ^18^O and δ^2^H ‰ vs. V-SMOW, respectively) were analyzed by Cavity Ring-Down Spectroscopy (CRDS) using a Picarro L2130-i instrument (analytical errors: δ^2^H ± 1.5 ‰ and δ^18^O ± 0.3 ‰ vs. V-SMOW).

The suspended load (TSS) was determined using the gravimetric method, i.e., water samples were filtered at 0.45 µm using cellulose acetate filters, dried (at 50 °C for 45 min in an oven), and weighed before and after filtration.

### Drainage basin structure and sub-basin identification

**Fig. 2:**
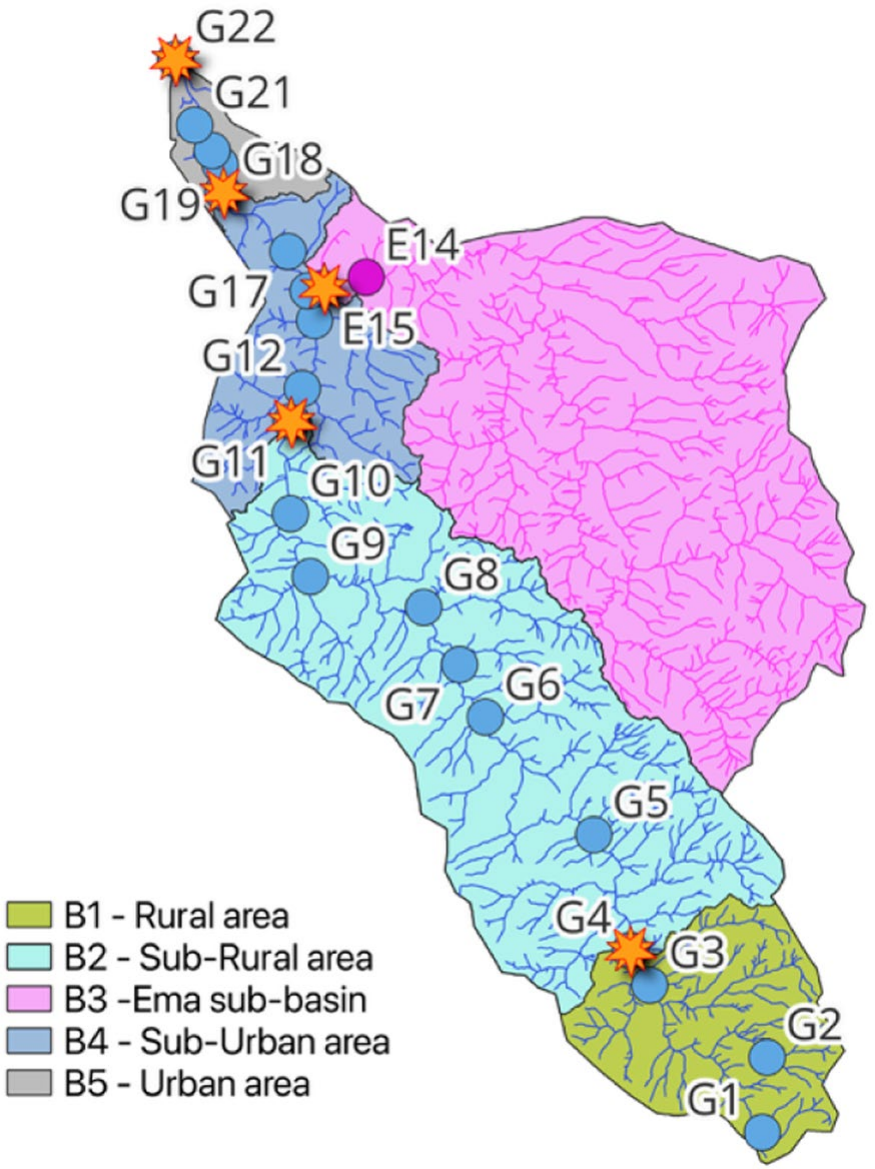
Watersheds of the sub-basins considered in this study. Water sampling points and control sites (orange stars) are also reported

River networks act as sentinels of their drained catchments (Li et al., 2022). Relevant studies have shown that there is a significant correlation between water quality and land use (Chen et al., 2020; Hariwati et al., 2026; Zhang et al., 2025), highlighting that areas with land uses associated with human activities tend to exhibit higher concentrations of water pollutants. In this study, we test the hypothesis that landscape influences on water quality are scale-dependent by dividing the GRB into five sub-basins (B1, B2, B3, B4, and B5; Fig. 2), considering five sampling points (G4, G11, E15, G18, and G22) as the pour point (outlet) of their corresponding contributing area. The subdivision of the basin was carried out based on land use, in order to identify a rural basin (B1), a sub-rural basin (B2), a sub-urban basin (B4), and an urban basin (B5). Separately, the sub-basin of the Ema stream (B3) was also identified, which can be classified as suburban. Each sub-basin was delineated in QGIS using the 10 m-resolution Hydrographic Digital Terrain Model (HDTM) provided by the Tuscany Region (Open Geodata-Regione Toscana). HDTM pre-processing was conducted using *WhiteboxTools* to fill spurious depressions and compute flow direction and flow accumulation, enabling accurate definition of the contributing areas associated with each point. For each sub-basin, the percentages of land use categories (including urban areas, olive groves and vineyards, forests, crops, grasslands, mining areas, and greenhouses) and geological categories (including sandstones, carbonatic flysch, marly limestones, mudstones and calcilutites, basalts, peridotites and ophiolitic breccias, sands, and pebbles) were calculated.

## Results

### Chemico-physical parameters and geochemical major characterization of the Greve River’s waters

**Fig. 3:**
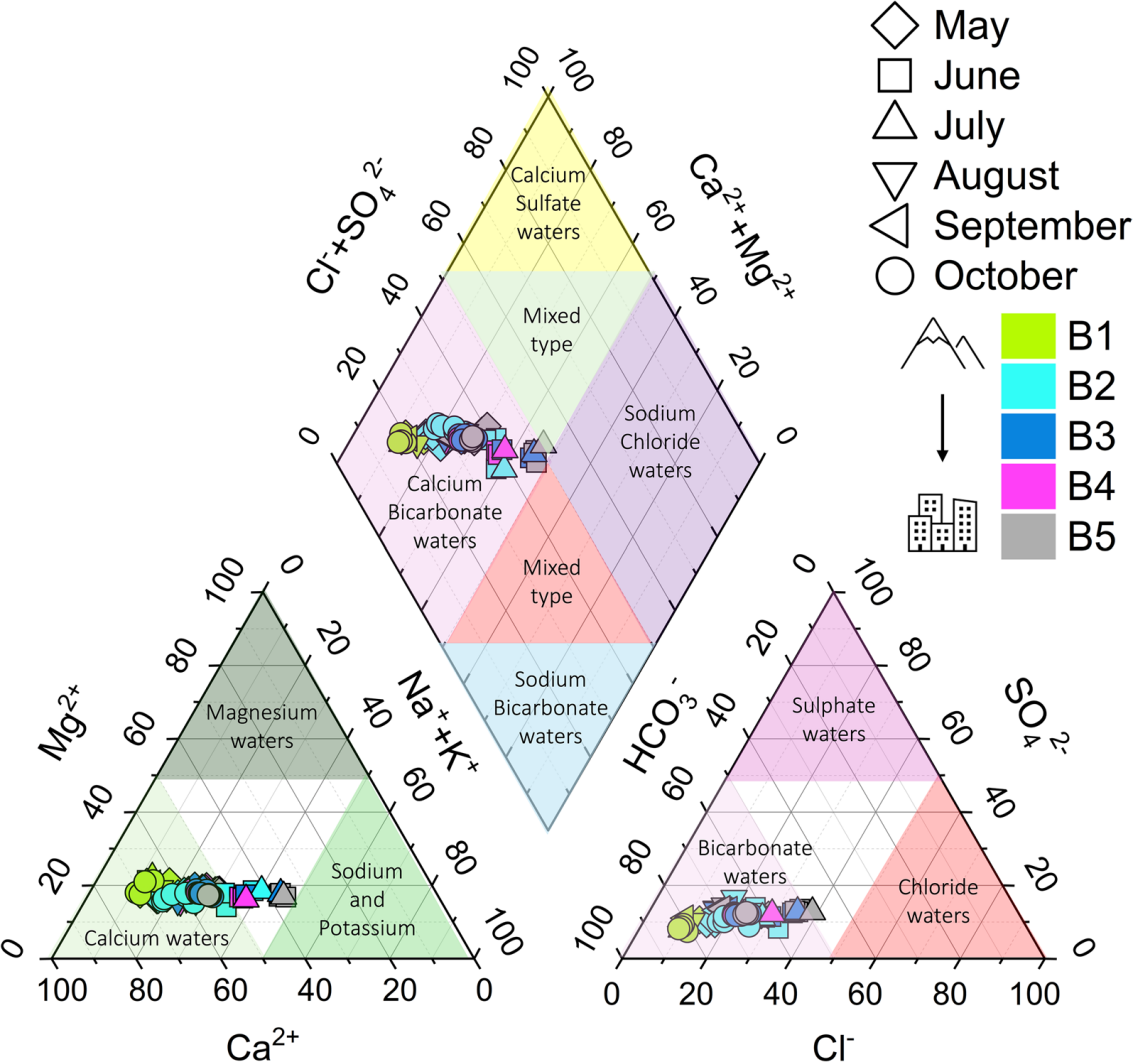
Piper diagram of anions and cations of Greve River waters, sampled from may to october 2022

This section presents the analytical results of water samples collected during the six sampling campaigns used to characterize the chemical composition of the GR. Data related to the suspended load is also shown. Over the entire monitored period (May–October 2022), suspended load concentrations (TSS) ranged from 0.001 to 0.265 g/L (Table 1), with the highest values recorded during the August campaign following the most intense rainfall event (see Supplementary Materials, Table S1 for the full dataset and Table S2 for detection limits). The minimum, maximum, and standard deviation of main chemico-physical parameters and main ion concentrations (in mg/L) are summarized in Table 1. The pH in Greve River waters varied between 7.56 and 8.74, while the oxidation–reduction potential (ORP) ranged between 65 and 345 mV. The total dissolved solids (TDS) value ranged between 273 and 699 mg/L. As indicated by the Piper Diagram (Fig. 3), calcium-bicarbonate is the predominant chemical facies, with a significant Na–Cl enrichment for samples collected in June and July. The highest variability was observed for Br^−^ (from 0.01 to 0.65 mg/L), NO_3_^−^ (from 0.14 to 21.5 mg/L), Na^+^ (from 9.92 to 80.9 mg/L), and K^+^ (from 1.11 to 11.3 mg/L) (Table 1), with mean concentrations higher in October (see Table S1 for the entire dataset). In contrast, HCO_3_^−^ (from 170 to 360 mg/L), SO_4_^2−^ (from 13.8 to 53.7 mg/L), Ca^2+^ (from 42.9 to 110 mg/L), and Mg^2+^ (from 8.86 to 19.7 mg/L) exhibited more homogenous and stable concentrations along the river and across seasons.

**Table 1:** Number of observations, Minimum (Min.), Maximum (Max.), and Standard deviation (SD) values of TSS, pH, ORP, main ions, TDS, trace elements, and minor components recalculated from the six sampling campaigns across the river

Parameter	Unit	No of observations	Min	Max	SD
TSS	g/L	38	0.01	0.265	0.05
pH	–	70	7.56	8.74	0.23
ORP	mV	70	65	345	79.5
TDS	mg/L	70	273	699	90.7
HCO_3_⁻	mg/L	70	170	360	41.1
F⁻	mg/L	70	0.11	2.64	0.35
Cl⁻	mg/L	70	14.8	120	25.00
Br⁻	mg/L	70	0.01	0.65	0.08
NO_3_⁻	mg/L	70	0.14	21.5	5.41
SO_4_^2^⁻	mg/L	70	13.8	53.7	9.62
Ca^2^⁺	mg/L	70	42.9	110	14.6
Mg^2^⁺	mg/L	70	8.86	19.7	2.73
Na⁺	mg/L	70	9.92	80.9	19.5
K⁺	mg/L	70	1.11	11.3	2.58
V	µg/L	70	0.32	3.62	0.78
Cr	µg/L	70	0.09	0.95	0.14
Mn	µg/L	70	0.81	86	19.4
Fe	µg/L	70	0.42	84.1	16.2
Ni	µg/L	70	0.33	4.23	1.07
Cu	µg/L	70	0.47	9.40	1.75
As	µg/L	70	0.12	2.1	0.51
Sb	µg/L	70	0.04	2.58	0.32
Pb	µg/L	70	0.02	2.6	0.40
NH_4_⁺	mg/L	70	0.01	1.35	1.44
NO_2_^−^	mg/L	70	0.005	0.72	0.18
COD	mg/L	30	1	100	19.7
P tot	mg/L	30	0.13	3.98	0.96

### Trace elements, minor components, and isotopic composition of GR waters

The concentration ranges of trace elements and minor components in Greve River waters are reported in Table 1. Regarding trace element contents, Mn and Fe exhibited the highest absolute variability, with concentration ranges of 0.81–86 µg/L and 0.42–84.1 µg/L, respectively (Table 1). Other elements, such as V (0.32–3.62 µg/L), Cr (0.09–0.95 µg/L), Cu (0.47–9.40 µg/L), and Sb (0.04–2.58 µg/L), showed lower variability across the river and throughout the entire monitored period. COD and P_tot_ reached higher values compared to NH_4_^+^ and NO_2_^−^, particularly during drought months (see Table S1 for the complete dataset).

Between May and October 2022, the isotopic composition of Greve River waters ranged from -41.9 to − 22.1 ‰ versus V-SMOW for δ^2^H and from − 7.49 to − 4.18 ‰ versus V-SMOW for δ^18^O. During drought periods (June and July), the river water was enriched in heavy isotopes (^18^O and ^2^H) (Table S1).

### Sub-basin land-use and lithological classes

The contribution area (km^2^) of (i) land-use and (ii) lithological classes was computed for each sub-basin (Fig. 2). This computation was carried out using the zonal statistics tool in a GIS environment (QGIS). Land use and geological layers were provided by the Tuscany Region (Open Geodata-Regione Toscana). The original thematic layers were reclassified by aggregating categories with similar characteristics into broader classes in order to improve interpretability (Figs. 1b, c), as the original datasets contained a higher level of detail than required for the scope of this study.

These results were then transformed into a percentage of contribution (Table 2). The land use categories considered are (Table 2, upper part): (i) urban areas, (ii) olive groves and vineyards, (iii) woods, (iv) crops, (v) grasslands, (vi) mining areas, (vii) plant nurseries and greenhouses. For the geology, we considered (Table 2, lower part): (i) sandstones, (ii) carbonatic flysch and marly limestones, (iii) mudstones and calcilutites, (iv) basalts, peridotites, ophiolitic breccias, and (v) sands and pebbles.

**Table 2:** Spatial changes of land use (upper part) and geological categories (lower table) across the five sub-basins delineated in the study area

Categories	Sub basins				
	B1 (G1-G4)	B2 (G5-G11)	B3 (E14-E15)	B4 (G12-G18)	B5 (G18-G22)
*Land use*					
Urban areas	3.2	3.8	3.36	3.99	5
Olive grows and vineyards	12.9	18.3	36.4	29.8	29
Woods	54.8	46	31.5	36.6	36
Crops	19.4	23.5	21.5	22.5	23
Grasslands	12.9	7.6	6.35	6.49	6
Mining areas	0	0.4	0.05	0.22	0
Plant nurseries and greenhouses	0	0	0.03	0.01	1
*Geology*					
Sandstones	84	38	38	37	36
Carbonatic flysch and marly limestones	0	13	11	13	12
Mudstones and calcilutites	16	31	32	33	32
Basalt, peridotites, ophiolitic breccias	0	2	2	1	1
Sand and pebbles	0	11	11	11	10
Detrital sediments	0	5	6	5	9

**Fig. 4:**
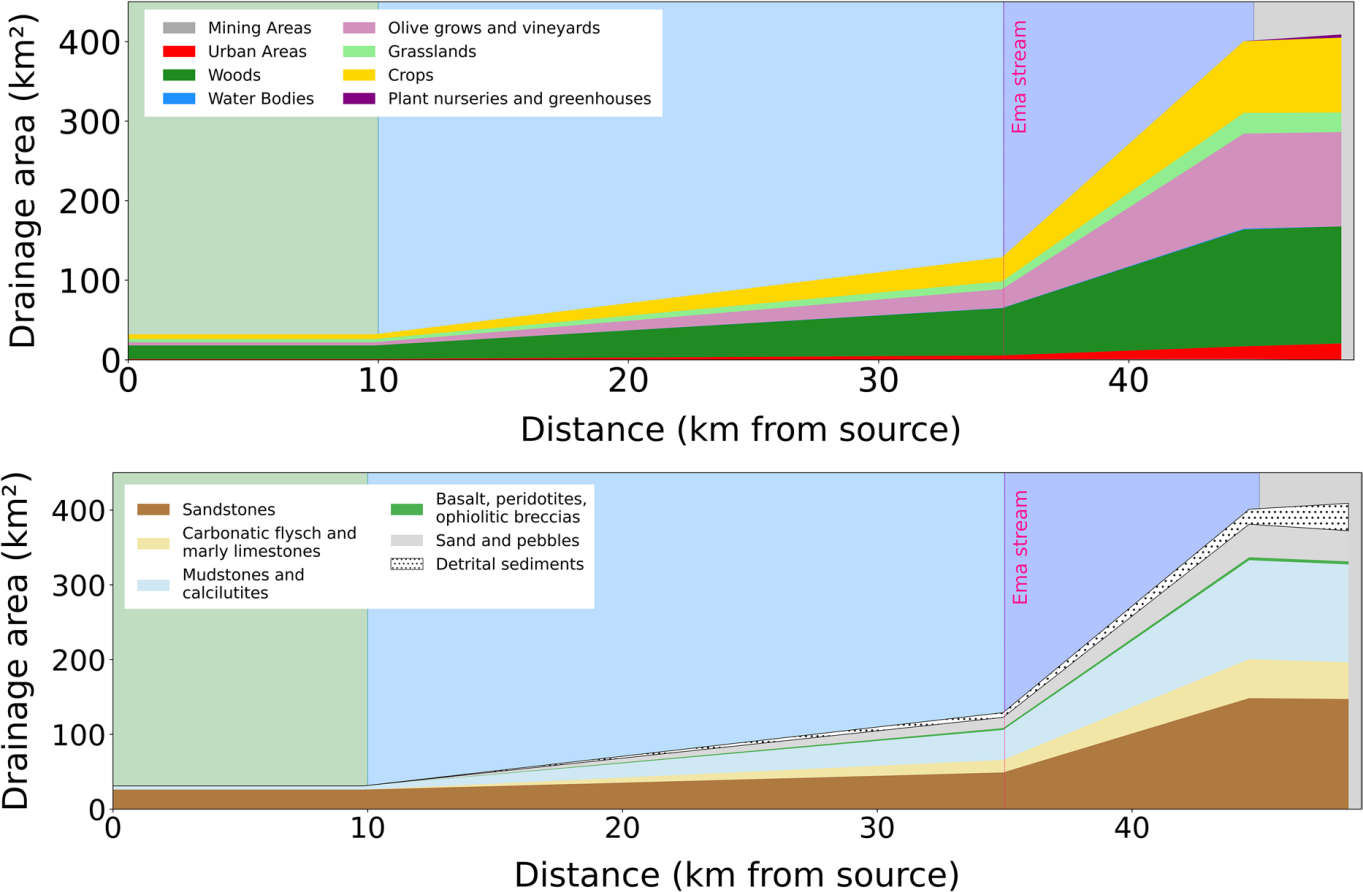
Streamgraph of land use (upper part) and geological categories (lower part) characterizing the different drainage areas along the Greve River. Coloured background panels indicate the identified sub-basins (B1, B2, B4, B5)

We then recalculated the drainage area for each category in each sub-basin and plotted it against distance (Fig. 4). This provides an overview of how the contributions of different land use or geological categories vary along the GR, from source to mouth.

## Discussion

### River source attribution based on isotopic and geochemical evidence

**Fig. 5:**
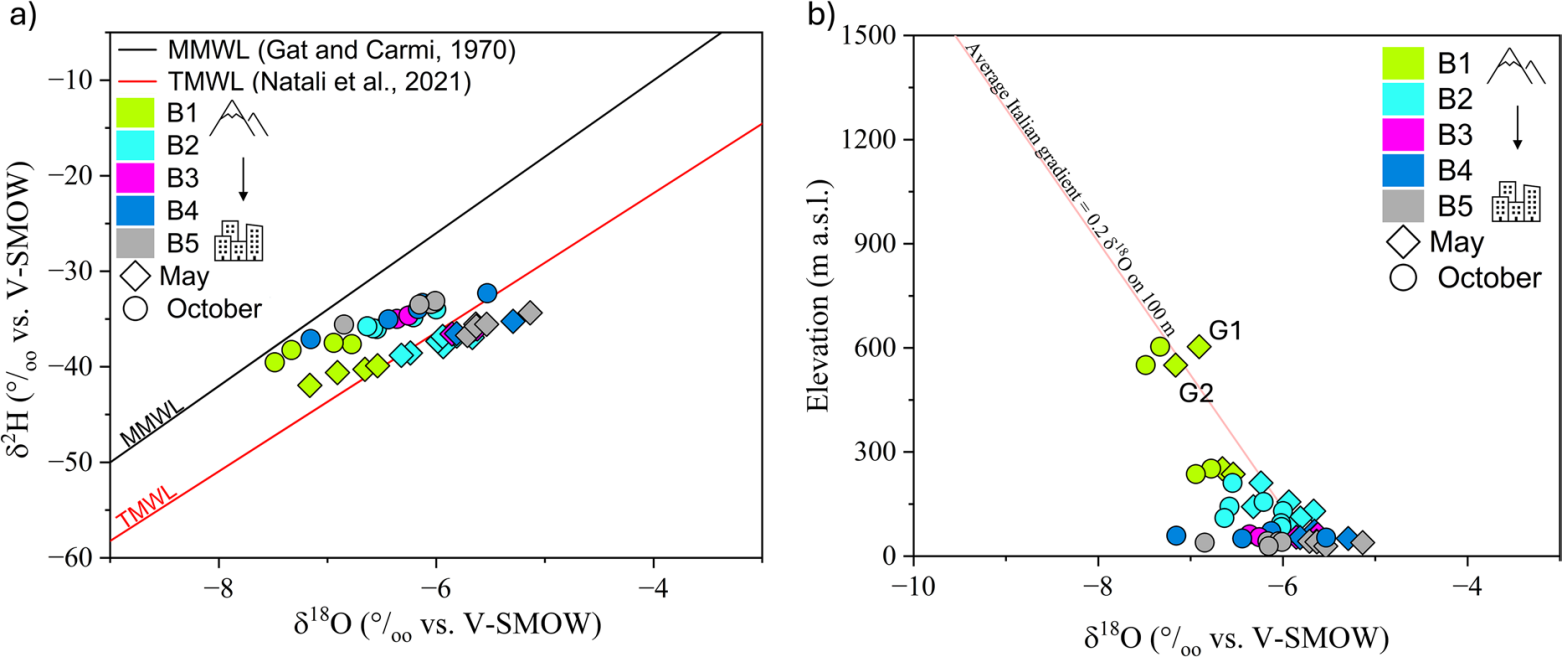
**a** δ^2^H versus δ^18^O biplot of water samples collected during May and October 2022, showing the Mediterranean Meteoric Water Line (MMWL; Gat & Carmi, 1970) and the Tuscany Meteoric Water Line (TMWL; Natali et al., 2021). Colours indicate sub-basins, and symbols distinguish sampling periods. **b** δ^18^O versus elevation (m a.s.l.) along the Greve River, compared with the mean Italian isotopic altitude gradient (Longinelli & Selmo, 2003)

Isotopic data of GR waters from the two complete sampling campaigns, as well as the Mediterranean Water Line (MMWL; Gat & Carmi, 1970) and the Tuscany Meteoric Water Line (TMWL; Natali et al., 2021) are reported on the δ^2^H versus δ^18^O biplot (Fig. 5a). Waters collected in May and October, respectively, display two parallel alignments with a different slope relative to those of MMWL and TMWL that delimit an area including almost all the samples. Such isotopic features were likely produced to the combination of different causes: (1) the decreasing altitude of the catchment areas feeding the different sections of the river from the mountain to the valley in both seasons (Liu et al., 2024; Xiao et al., 2025), (2) isotope fractionation due to water evaporation from the river surface (Kim & Lee, 2011), and (3) a heavier meteoric source occurring in May relative to that sourcing the river in October likely caused by a different (i) air temperature, typical of the two seasons, and/or (ii) meteoric water (clouds) provenance (Giustini et al., 2016). It is worth noting that waters from the lower part of the basin (B4 and B5; Fig. 5a) showed the most pronounced ^18^O- and ^2^H-enrichments, suggesting that the flat morphology and the relatively high temperatures of the strongly urbanized area, including these two sub-basins, favoured the isotopic fractionation due to evaporation (Gallart et al., 2024; Yang & Wang, 2024). As shown in Fig. 5b, where the theoretical isotopic gradient with altitude of the meteoric rainwater for precipitation in Italy is reported (− 0.22‰ per 100 m; Longinelli & Selmo, 2003), river water samples from the highest elevations (G1 and G2 in the sub-basin B1) indicate a recharge area at approximately 600 m a.s.l. (Fig. 5b), whereas the samples from areas towards the valley were enriched in δ^18^O, consistent with the mean sub-basin elevations (from B1: 513 m to B5: 280 m a.s.l.), confirming the effect of a decreasing recharge altitude indicated in Fig. 5a.

**Fig. 6:**
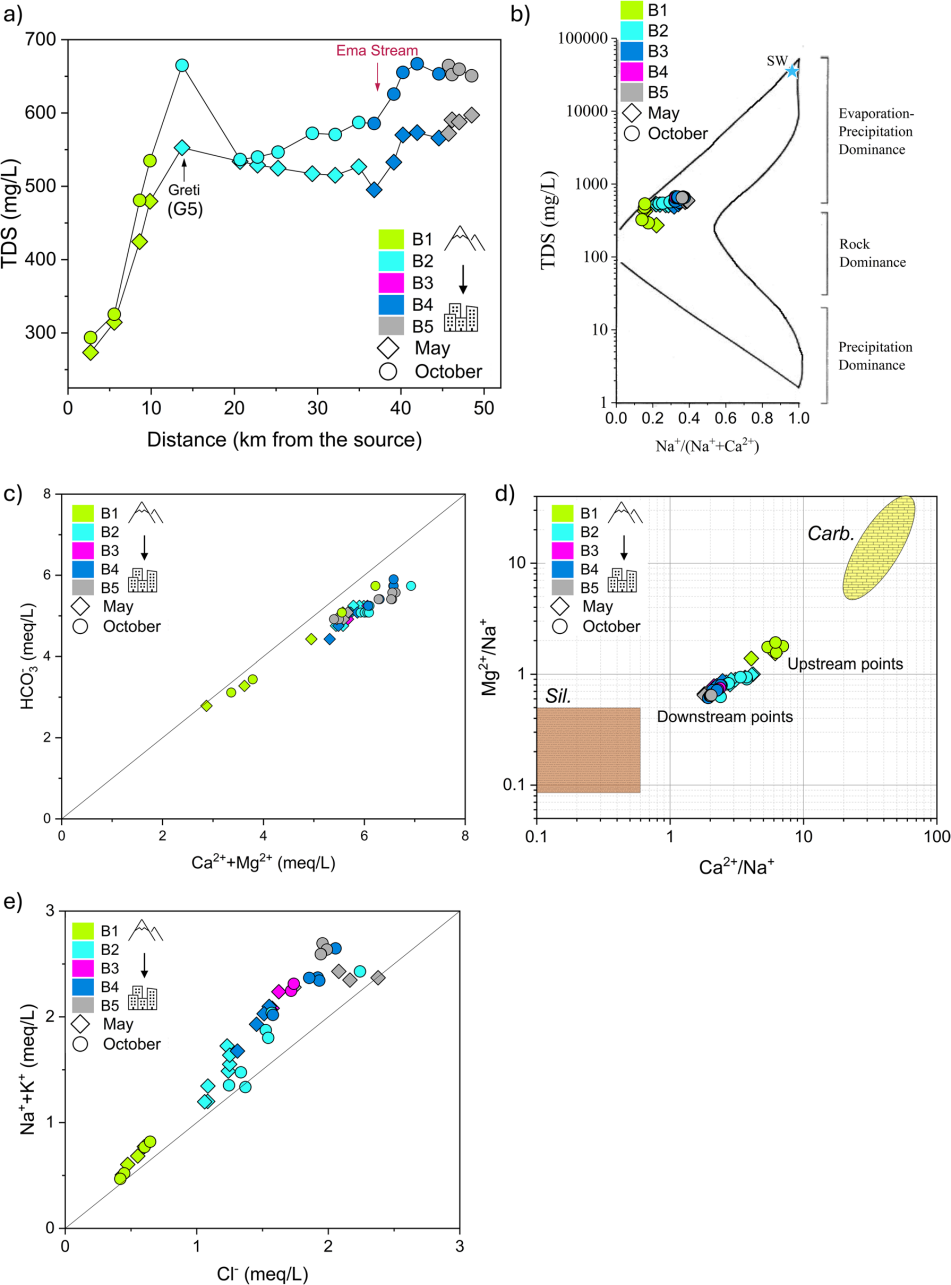
Major ion geochemistry of the Greve River during May and October 2022. **a** Spatial distribution of TDS. **b** Gibbs diagram. **c** HCO_3_^−^ versus Ca^2+^  + Mg^2+^. **d** Ca^2+^/Na^+^ versus Mg^2+^/Na^+^ endmember diagram after Gaillardet et al. (1997). **e** Na^+^ + K^+^ versus Cl^−^. Stoichiometric 1:1 dissolution lines are shown in panels (**c**) and (**e**)

A spatial trend can also be observed in the TDS values (Fig. 6a), which exhibit a progressive increase from the river source to mouth, with slightly higher values in October relative to May (Fig. 6a). Such spatial patterns are consistent with the theoretical Gibbs model (Fig. 6b), which describes the downstream evolution of river water chemistry due to cumulative solute inputs. During both sampling campaigns, a TDS peak value was recorded at G5 (Fig. 6a), near the locality of Greti (Greve in Chianti, Fig. 1a), a rural area with intensive agricultural activity, where historical data have already documented similar chemical variations (Nisi et al., 2008). The TDS trends were also influenced by inflow from the Ema stream, which accentuated the increasing TDS pattern of the GR sub-basin entering the urban area (B4; Fig. 6a). The Ca^2+^–HCO_3_^−^ composition of all the waters (Fig. 3), was likely mostly dictated by carbonate weathering (Fig. 6b). However, the Ca^2+^ + Mg^2+^ concentrations were slightly exceeding relative to the stoichiometric ratio with HCO_3_^−^ (Fig. 6c), suggesting that carbonate dissolution was not the only Ca^2+^ and Mg^2+^ source. This excess of cations may also be attributed to the alteration of silicate minerals, which can release additional Ca^2+^ and Mg^2+^ into solution**.** Although the weathering of silicate minerals proceeds more slowly relative to that of carbonate rocks (Bufe et al., 2021), downstream waters, being related to longer hydrological pathways relative to upstream ones, typically show relatively high silicate-derived solutes. Following the model of Negrel et al. (1993), the Mg^2+^/Na^+^ and Ca^2+^/Na^+^ ratios were used to recognize solute contributions from silicate or carbonate rocks (Fig. 6d), whose endmember compositions were defined based on global lithotype averages (Gaillardet et al., 1997). As expected, downstream waters, especially those from the B2 sub-basin onward, tend to approach the silicate endmember more than upstream waters. Silicate weathering likely also contributed to the Na^+^- and K^+^-excess relative to the stoichiometric ratio with Cl^−^ (Fig. 6e).

**Fig. 7:**
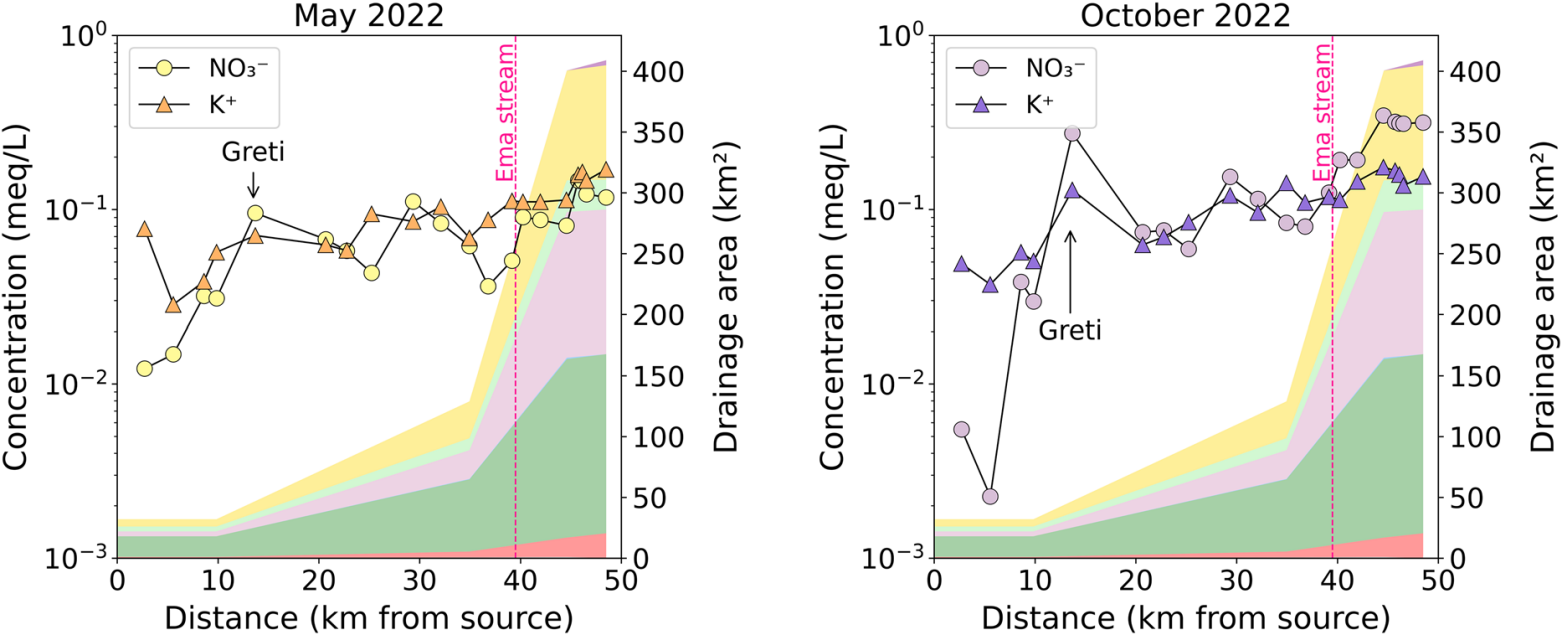
Spatial variation of NO_3_^−^ and K^+^ along the Greve River during May (left panels) and October (right panels) 2022. Land-use categories and corresponding drained sub-basins are shown in the background. The confluence with the Ema stream is indicated by the pink dashed line

As previously mentioned, the main agricultural activities in the GRB consist of olive and grape cultivations (Fig. 1b), which play a key role in sustaining the local economy. Fertilizers, herbicides, and other pesticides are commonly used within the basin to enhance and increase crop productivity (Adebanjo-Aina et al., 2025). Nitrogen, as well as potassium, is one of the most important nutrients for crop production (Hina, 2024), as it plays a crucial role in plant growth and development. The land use classification of the GRB shown in Fig. 4, i.e., crops, olive groves, vineyards, and urban areas, suggests an increased input of pollutants containing these two elements in the lower stretch of the river. Accordingly, NO_3_^−^ and K^+^ in the GR waters were positively correlated (Fig. 7), reflecting the high agricultural pressure in this area, with concentration peaks near Greti (G5), particularly for NO_3_^−^ (Fig. 7). It is worth nothing that the NO_3_^−^ and K^+^ concentrations significantly increased after the confluence with the Ema River (Fig. 7), in agreement with the land use in the Ema sub-basin (B3 in Table 2), which is dominated by olive groves and vineyards (36.4%), as well as crops (21.5%).

**Fig. 8:**
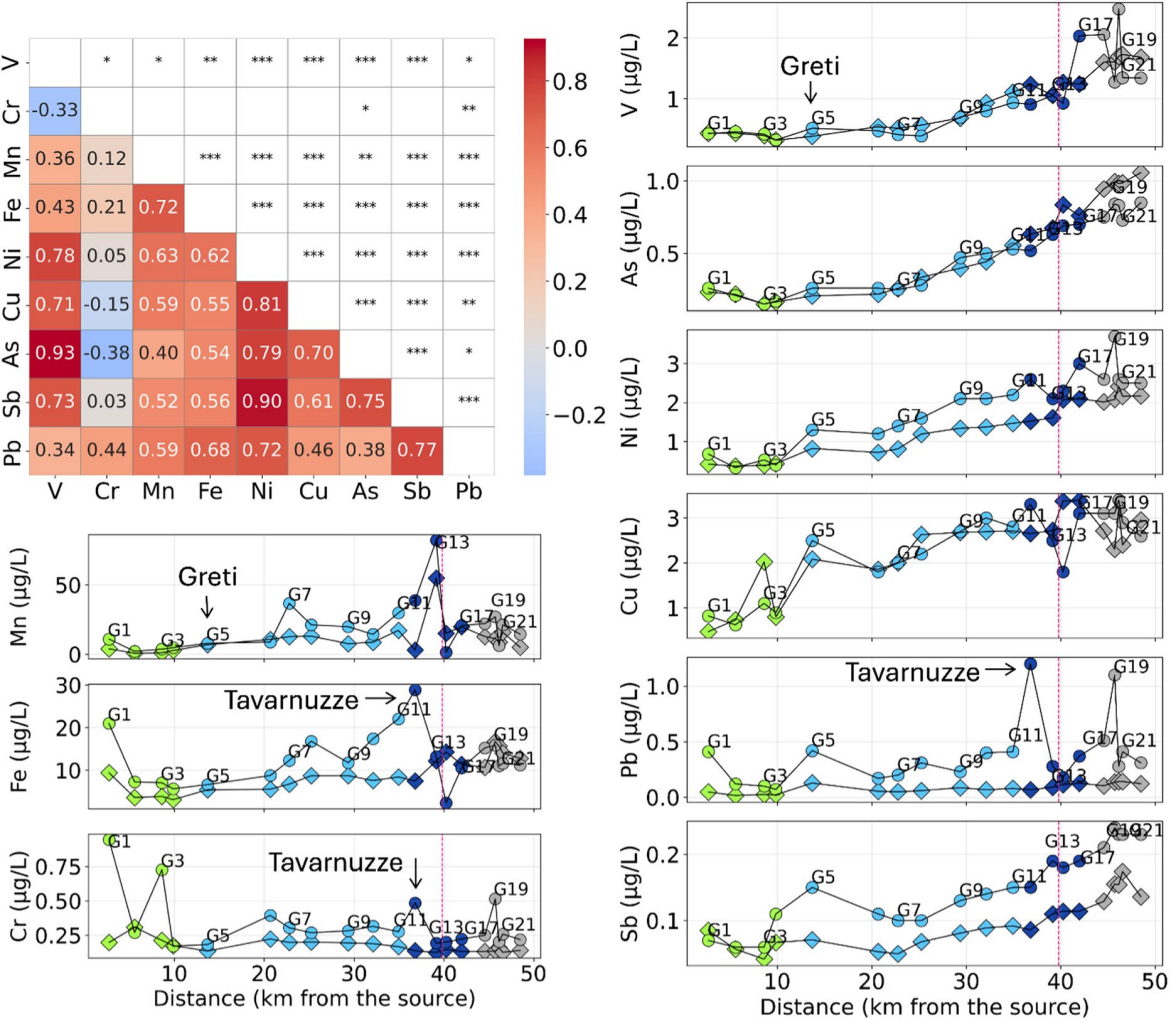
Spearman correlation matrix for the entire dataset, showing correlation coefficients and their associated p-value significance levels. The spatial variability of each trace metal is shown using the same colors and symbols as in the previous figures

Runoff from urban and agricultural landscapes is a well-known contributor to trace elements in surface waters, alongside the influence of regional geology (Richardson, 2020). To explore potential differences between natural and anthropogenic influences, a correlation matrix of trace elements (V, Ni, Cu, As, Sb, Cr, Mn, Fe, and Pb), together with their spatial variation along the river during May and October 2022, was constructed and presented in Fig. 8. It is noted that the correlation matrix integrates samples from two hydrological periods (May and October; Fig. S1) and therefore represents an overall pattern of co-variation rather than phase-specific relationships. Positive correlations were observed among the so-called “Traffic Related Elements” (TREs), i.e., Ni, Sb, As, Cu, V, and Pb, suggesting potential common anthropogenic sources and a similar transport behaviour. In particular, urban runoff and traffic-derived emissions may contribute to the observed spatial increase in (i) Cu and Sb from vehicle wear and brake abrasion, (ii) Ni, As, and V from combustion processes and tire wear (Shajib et al., 2019; Wagner et al., 2018; Yang et al., 2016) from the GR source to the mouth, where the main urban centers are located (Fig. 1b). Conversely, Pb exhibit an almost stationary distribution along the GRB which may explain the weaker correlations and lower significance observed with the other TREs (Fig. 8). Two exceptions were identified at sites G12 (Tavarnuzze) and G19 in October, both associated with a slight increase in Cr and, in the case of G12, also in Fe (Fig. 8). Although these peaks do not indicate a significant Pb input (ca. 1 µg/L), they may have been related to local (and likely transient) sources, likely linked to traffic jam due to the nearby FI-SI highway at G12 (Fig. 1) (Shajib et al., 2019), and a more diffuse urban pressure at G19, where peaks in Pb were accompanied by an increase of the other TREs (Fig. 8). Greti site (G5 in Fig. 8), where a strong agricultural pressure was evidenced by high TDS values (Fig. 6a) and anomalous concentrations of NO_3_^−^ and K^+^ (Fig. 7), exhibited peaks in (i) Cu, commonly associated with pesticide and fungicide application (Burandt et al., 2024; Fagnano et al., 2020), (ii) Sb and (iii) Pb and Ni (though to a lesser extent), which may reflect a combination of agricultural machinery wear and diffuse anthropogenic inputs (Wagner et al., 2018).

Thought the highest concentrations of V and As in the urban sub-basin B5 (Fig. 8) were likely caused by the enhanced anthropogenic inputs characterizing this zone, we cannot exclude that the relatively low increases of these two elements in sub-basins B1, B2, and B4 (Fig. 8) may reflect geogenic contributions associated with clay-rich lithologies (mudstones and calcilutites; Fig. 1c). These rocks typically act as adsorption substrates for As and V and promote their release as oxyanions through desorption processes under mildly alkaline and oxidizing conditions (Abernathy et al., 2022; Mikkonen et al., 2019; Peel et al., 2022). However, all concentrations of TREs remained well below the threshold values established by the Italian drinking water regulation (D.Lgs. 18/2023) and were also lower than regional background levels, as defined by the mean concentrations measured across the entire Arno River basin (Nisi et al., 2008) (see Table S2 for reported limits).

A geogenic source, involving desorption from clay-rich lithologies (mudstones and calcilutites in Fig. 1c) and mobilization during groundwater circulation via the reductive dissolution of Fe and Mn oxides, is also likely responsible for the occurrence of Fe and Mn, which were significantly correlated (r^2^ = 0.72). Noteworthy, Mn concentrations at point G13 were the highest measured in both seasons (Fig. 8). Both values (55 µg/L in May and 82 µg/L in October) exceeded the admissible concentration for this element in drinking water (50 µg/L; Table S2), set by Italian Legislative Decree 18/2023 (D.Lgs.^18^/, 2023, 2023). Such high Mn concentrations were already highlighted by previous investigations focusing on domestic wells near this sampling site, within the underlying Foredeep Sandstones aquifer of Northeastern Tuscany-Apennine Dorsal Zone, although the origin of these Mn anomalies remains debated (ARPAT, 2023). The localized enrichment observed at G13 may therefore be influenced by interactions with groundwater inputs, potentially reflecting mixing processes between surface water and Mn-rich groundwater from the underlying aquifer system.

### Seasonal variations in GRB: effects of prolonged drought and extreme first- flush event

In 2022, Italy was affected by air temperatures exceeding those recorded in 1991–2020 (on average + 1.23 °C; SNPA, 2023), and, more generally, this year was the hottest and driest since 1961. Such anomalous conditions led to early soil moisture deficits and low river flows across the Mediterranean (Bianchini et al., 2025; Biella et al., 2025; Chelli, 2023; SNPA, 2023). Annual cumulative precipitation in Italy during 2022 was 22% lower than the climatological average (Bianchini et al., 2025), resulting in pronounced hydrological stress across river systems. Within this context, the temporal evolution of the GR clearly reflects such hydrological stress, with its isotopic and chemical composition consistent with the expected effects of prolonged drought followed by a first-flush event. Under these conditions, the dominant processes regulating riverine biogeochemistry typically shift from in-stream physical–chemical processes prevailing during low-flow conditions (e.g., sediment–water exchange processes, evapoconcentration, and chemical interactions involving dissolved solutes) to a predominance of externally derived inputs (both natural and anthropogenic) associated with enhanced hydro-mechanical transport during the first-flush event (Mamun et al., 2020; Maniquiz-Redillas et al., 2022).

**Fig. 9:**
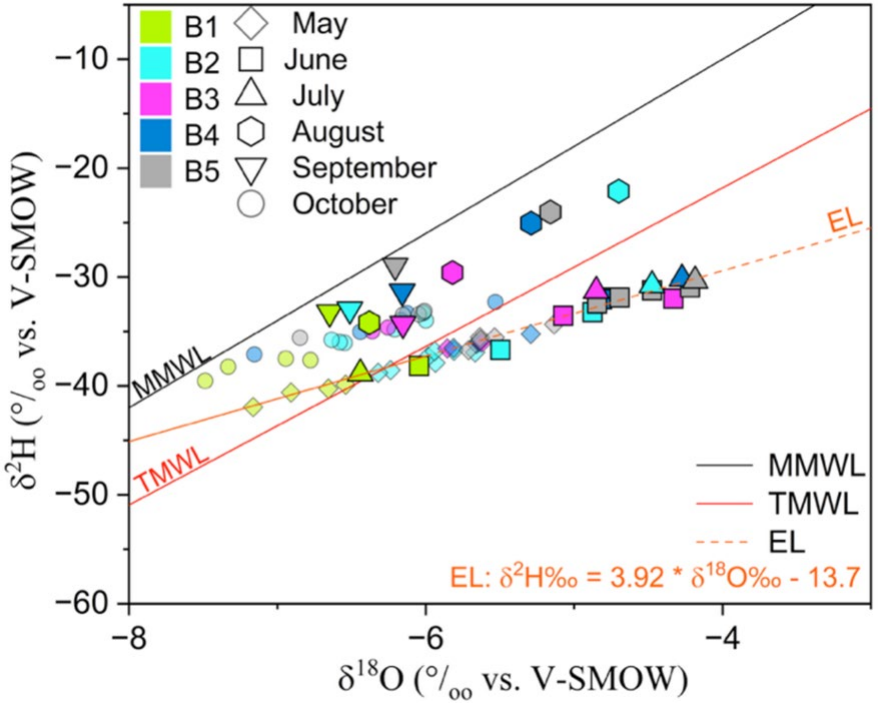
δ^2^H versus δ^18^O biplot of river water samples collected during the entire monitored period (May–October), together with Mediterranean Meteoric Water Line (MMWL, Gat & Carmi, 1970), and Tuscany Meteoric Water Line (TMWL, Natali et al., 2021). The Evaporation Line (EL: δ^2^H = 3.92 × δ^18^O–13.7) of the driest samples is also shown

**Fig. 10:**
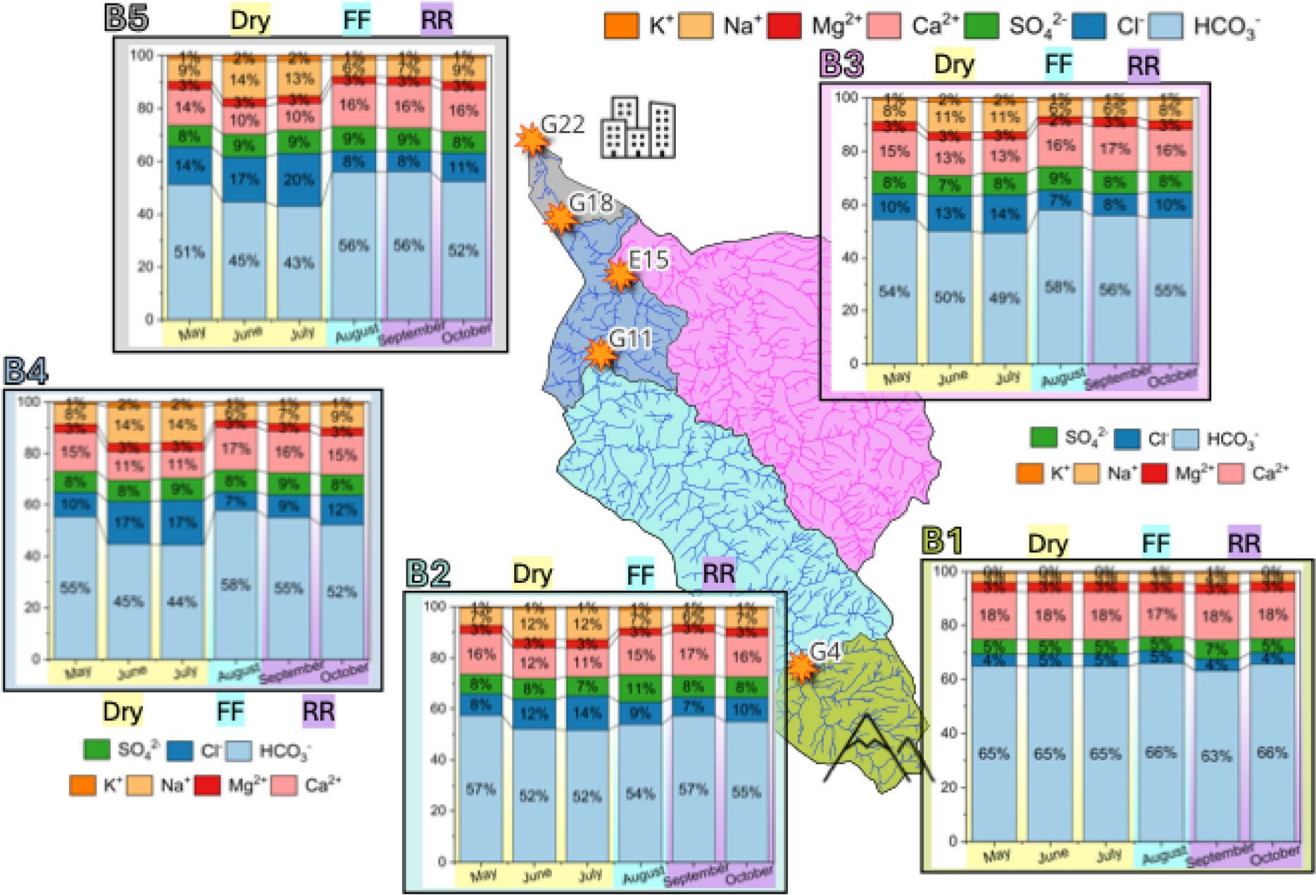
Percentage distribution of the major ions at the five points (G4, G11, E15, G18, and G22, corresponding to B1, B2, B3, B4, and B5, respectively) shown as 100% stacked columns. Each column represents the monitored month, and the coloured segments indicate the relative contribution (%) of the individual ions. Background shading indicates hydrological conditions: Dry (yellow), First Flush (FF, light blue), and Regular Rainfall (RR, purple)

**Fig. 11:**
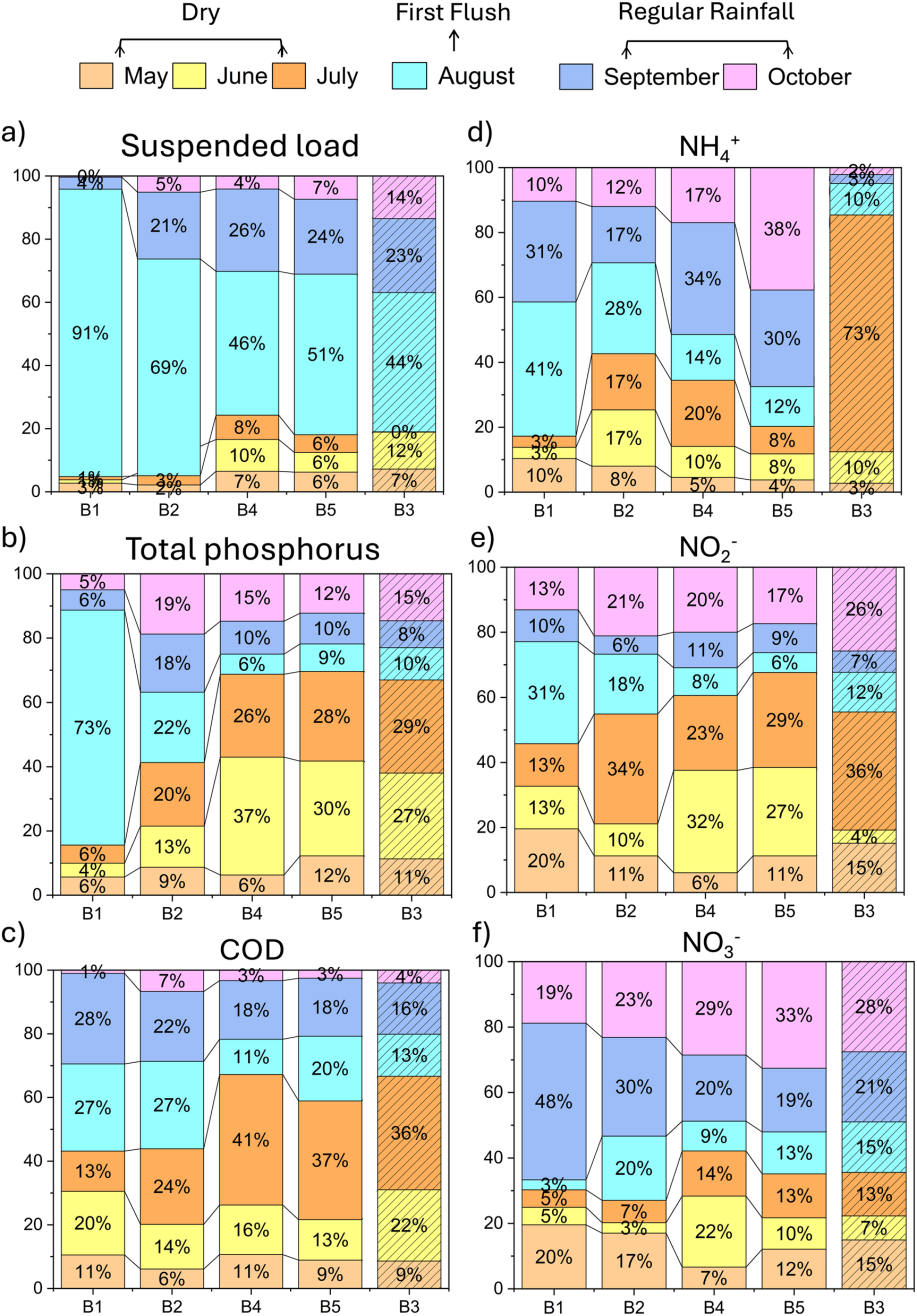
Relative distribution of **a** suspended solids (TSS), b total phosphorus (P_tot_), **c** COD, and nitrogen species (**d** NH4^+^, **e** NO_2_^−^, **f** NO_3_^−^) at the five representative sampling sites (G4, G11, E15, G18, and G22; corresponding to sub-basins B1–B5). Results are shown as 100% stacked columns, where coloured segments represent the percentage contribution of each monitored month

Accordingly, the monitored months were grouped into three hydrological phases, based on isotopic composition and major ion chemistry (which are discussed in detail in the following section; Figs. 9, 10, 11), as follows: (i) Dry (May–July), characterized by prolonged drought, with an average discharge recorded at the closing station of ~ 0.60 m^3^/sec^−1^ and a mean rainfall of 0.24 mm (SIR – Regione Toscana, 2025; Fig. S1), (ii) First Flush (August), representing the intense rainfall event that occurred after the drought (mean discharge of ~ 1.66 m^3^/sec^−1^ and average rainfall of 3.61 mm; Fig. S1), and (iii) Regular Rainfall (September–October), characterized by lighter rainfall events and more stable wet conditions (mean discharge of ~ 1.22 m^3^/sec^−1^ and average rainfall of 2.30 mm; Fig. S1).

The hydrological classification was further statistically evaluated using non-parametric Kruskal–Wallis tests followed by Dunn’s post-hoc comparisons (Table 3). Results confirmed that rainfall (obtained from the Regional Hydrological Service; SIR—Regione Toscana, 2025) and suspended solids (TSS) significantly differ among all hydrological phases (*p*-value < 0.01; Table 3), supporting the robustness of the phase separation and highlighting the strong hydrological control on particulate dynamics. In contrast, most dissolved parameters (TDS) did not exhibit significant phase-dependent variability (*p*-value > 0.05; Table 3), indicating a more conservative behaviour dominated by background processes. Among trace elements, Cr, Fe, and Cu showed significant differences between hydrological phases, whereas Sb exhibited only marginal trends, and V, Mn, Ni, As, and Pb did not vary significantly (Table 3).

**Table 3:** Results of the Kruskal–Wallis test and Dunn’s post-hoc comparisons

Group	Variable	Kruskal significance	Main Dunn result	Interpretation
Hydrology	Rain	*p* < 0.001	Dry ≠ first flush, first-flush ≠ regula rain	Phases well separated
Hydrology	TSS	*p* < 0.01	Dry ≠ first flush, first-flush ≠ regula rain	First flush peak
Trace metals	Cr	*p* < 0.01	Dry ≠ first flush	Event-driven
Trace metals	Fe	*p* < 0.01	Dry ≠ first flush	Particulate-associated
Trace metals	Cu	*p* < 0.01	Dry ≠ first flush, first-flush ≠ regula rain	Multi-phase response
Trace metals	Sb	~ *p *≈ 0.04	Marginal	Weak trend
Trace metals	V, Mn, Ni, As, Pb	Not significative	No significant differences	Geogenic/stable
Nutrients	NO_3_⁻, NH_4_⁺	Not significative	No significant differences	Conservative behavior
Bulk parameter	TDS	Not significative	No significant differences	Baseline control

During the Dry phase (average temperatures of 24.3 °C and 26.6 °C in June and July, respectively; SIR—Regione Toscana, 2025), isotopic enrichment of δ^18^O and δ^2^H was observed in GR waters under evaporative conditions, as indicated by the evaporation line (EL: δ^2^H = 3.92 × δ^18^O − 13.7; Fig. 9) (Bianchini et al., 2025). The slope of the evaporation line (b = 3.92; Fig. 9) is lower than that of the TMWL (b ≈ 7.3; Natali et al., 2021), indicating evaporative fractionation under non-equilibrium conditions, consistent with progressive water loss during the dry period. On the other hand, during the First-Flush event, river samples plot between MMWL and TMWL (Fig. 9), reflecting the arrival of new, poorly evaporated water, characteristic of summer/warm-season rainfall (Tian et al., 2018; Valdivielso et al., 2024). Relative to August (Fig. 9), waters collected in September and October (Regular Rainfall) exhibited lower δ^2^H and δ^18^O values, due to the relatively lower temperature of the meteoric waters during this period (Valdivielso et al., 2024).

The shift in dominant processes that regulate river biogeochemistry is reflected in the chemical composition of GR. As shown in Fig. 10, the main chemical changes recorded in June and July relative to the previous period include: (a) a decrease in HCO_3_^−^ and Ca^2+^, likely due to reduced mechanical water–rock interaction under low-flow conditions; (b) an increase in Cl^−^ and Na^+^, particularly in the downstream basins (B2, B3, B4, and B5; Fig. 10) where channel geometry and morphology favour stagnant conditions and evaporation, as also indicated by the isotopic signatures of the water samples from these areas (Fig. 9); (c) an increase in K^+^, especially in the downstream basins where these stagnant conditions combined with high temperatures, could explain its accumulation (Fig. 10). Suspended load (TSS) remained relatively low during low energy hydrological conditions (Dry phase; Fig. 11a), indicating limited erosive activity and sediment transport (Mosley, 2015; van Vliet et al., 2023). The slight downstream increase in TSS (Fig. 11a) was likely caused by (i) local inputs, given the increase of potential anthropogenic inputs, affecting the B4 and B5 sub-basins (Fig. 4), and/or (ii) enhanced sediment supply associated with the Ema stream (B3; Fig. 11a) inflow. Conversely, both P_tot_ and COD, closely associated with organic material, progressively increased downstream (Fig. 11b, c), likely due to increasing anthropogenic pressure. Elevated COD values may result from evaporative concentration, accumulation of partially degraded matter, and production of autochthonous organic matter (Kuo et al., 2025). These processes are favoured by water temperature and nutrient availability, particularly phosphorus, which followed a trend similar to that of COD (Fig. 11b). Indeed, in the suburban and urban sub-basins (B4 and B5, respectively), the river receives tributaries that drain areas rich in anthropogenic potential sources of phosphorus, e.g., domestic wastewater and fertilizers from intensive cultivation (Figs. 1b, 5). After the First-Flush event, the regime shifted towards more dynamic conditions, when external inputs became dominant. Enhanced hydro-mechanical transport promoted the rapid mobilization of both natural and anthropogenic materials from surfaces (soil and anthropogenic infrastructures) exposed to dry deposition in the catchment basin. Statistical analysis (Table 3) confirmed that TSS varies significantly among hydrological phases, with markedly higher values during the first-flush event, highlighting the strong hydrological control on particulate mobilization. In this context, the observed variations in major ions (HCO_3_^−^, Ca^2+^, Cl^−^, and Na^+^; Fig. 10) suggest a shift in the balance between water–rock interaction and hydrological dilution/concentration processes, although these patterns were not statistically evaluated and should therefore be interpreted as indicative trends. The concomitant increase in P_tot_ and COD at the upstream sampling sites (Fig. 11b–c) suggests that phosphorus and organic matter were mostly transported in association with the particulate fraction during high-flow conditions.

During the dry phase, NH_4_^+^ concentrations were higher in downstream sectors (Fig. 11d), which may reflect the combined effects of reduced nitrification, longer residence times, soil nitrogen accumulation, and potential wastewater inputs in more impacted areas. Nitrite exhibited relatively elevated concentrations during low-flow conditions (Fig. 11e), consistent with reduced oxygen availability, as also indicated by COD levels (Fig. 11c; Fuchslueger et al., 2014; Hammerl et al., 2019; Leitner et al., 2017; Vázquez et al., 2025). The contribution of nitrogen species was particularly relevant in the Ema sub-basin (B3; Fig. 11d, e), likely related to the extensive vineyards and olive groves occurring in the area (Fig. 1b and Table 2).

During the First Flush event, NH_4_^+^ and NO_2_^−^ increased in upstream sub-basins (B1, B2), suggesting rapid mobilization of nitrogen stored during the dry period (Hammerl et al., 2019; Krüger et al., 2021; Leitner et al., 2017; Yang et al., 2015), whereas oxidation processes reduced the concentrations of these species in waters from downstream areas (Fig. 11d, e). With sustained rainfall in September and October, NO_3_^−^ dominated nitrogen dissolved species (Fig. 11f), consistent with increased nitrification after soil rewetting and nitrate leaching from agricultural soils (Hina, 2024). On the whole, these results demonstrate that nitrogen export was tightly controlled by the interaction between seasonal hydrological events and biogeochemical transformations in soils and agricultural landscapes, although their response to hydrological phases is less statistically pronounced than that observed for particulate-associated variables.

**Fig. 12:**
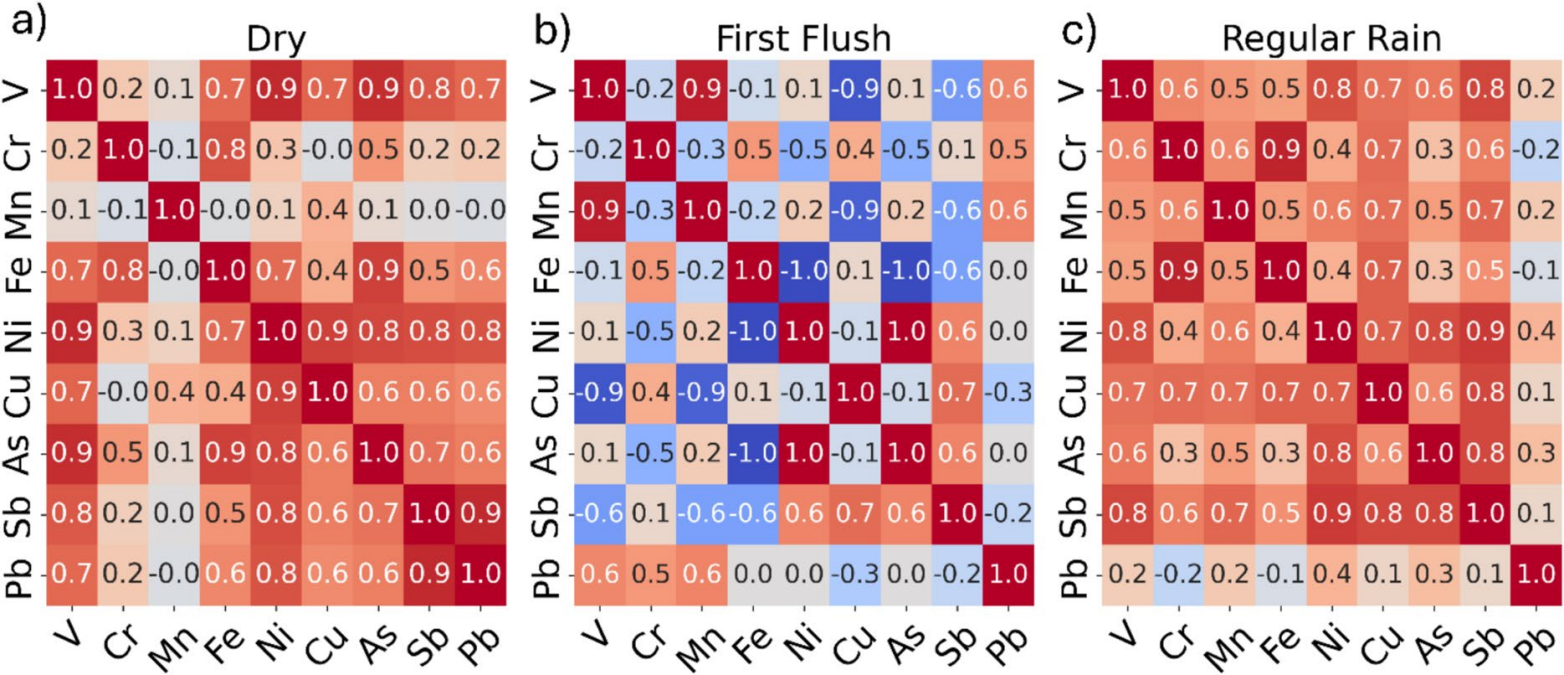
Spearman correlation matrices of dissolved trace elements in Greve River waters during the three identified hydrological phases: **a** dry period (May–July), **b** first flush event (August), and (**c** regular rainfall conditions (september–october)

Figure 12a–c shows the Spearman correlation matrices calculated for each hydrological phase. During the Dry phase (Fig. 12a), V, Fe, Ni, Cu, As, Sb, and Pb were strongly related, suggesting a dominant control of internal river processes under low-flow conditions. These relationships likely reflect the combined effect of: (i) hydrological accumulation due to prolonged low-flow, (ii) extended water–sediment interactions that promote the release of metals associated with fine particles (clay-rich lithologies; Fig. 1c), and (iii) accumulation of organic matter causing an oxygen demand, as indicated by COD and phosphorus levels (Fig. 11b–c). The latter favoured the dissolution of iron oxides and the co-release of adsorbed metals. Manganese appeared largely decoupled from these metals. Its behaviour is controlled by redox-driven mobility, as Mn oxides are readily reduced under mildly suboxic conditions, releasing soluble Mn^2+^ that does not strongly associate with particulate matter like Fe or V (Owings et al., 2021).

During the First Flush phase (Fig. 12b), correlation patterns among trace elements became more heterogeneous, indicating a shift in system organization under strong hydrological forcing. This correlation structure (Fig. 12b) may suggest the simultaneous contribution of multiple processes, including particulate-bound transport, dissolved-phase inputs, and spatially variable source contributions. In addition, the flushing event likely mobilized readily soluble metals from surface soils and anthropogenic deposits accumulated during the dry period, further contributing to the observed chemical variability (Kittlaus et al., 2025). Overall, these patterns indicate a transition toward a multi-source, hydrologically driven transport regime during the First Flush event.

During the Regular Rain phase (Fig. 12c), water runoff in the river catchment area gradually stabilized. The river flow was still increasing, but rainfall was less intense, and correlations among trace elements reorganized: some returned to be positive, while others remained weak (Fig. 12c). This indicates that the hydrological regime still controlled the trace element transport, but less impulsively than during the first flush. Strong associations persisted among Ni, Sb, As, Cu, and Fe, in relation to the local lithology (clay-rich formations and possibly metamorphic units; Fig. 1c) and anthropogenic inputs (i.e., agricultural activities and urban areas).

Overall, the Spearman correlation analysis indicates a progressive shift from a chemically structured system dominated by internal geochemical processes (Dry) to a more heterogeneous and hydrologically driven system (First Flush), followed by a partially stabilized regime, when hydrological and chemical influences coexisted (Regular Rain). These patterns are consistent with the hydrological phase classification and complement the statistically significant differences observed in suspended solids and selected trace elements across phases.

## Conclusions

This study demonstrates that the short-term geochemical variability in river systems exposed to strong rural–urban gradients is primarily driven by rapid changes in hydrological regimes. Particular attention was devoted to the extreme drought conditions that affected Europe during 2022, an event already recognized by several recent studies as one of the most severe hydrological anomalies of recent decades (Bianchini et al., 2025; Biella et al., 2025; Bonaldo et al., 2022; Montanari et al., 2023). However, while the impacts of the 2022 drought have been investigated in several European regions, comparable hydrogeochemical studies remain scarce for Central Italy, especially for smaller peri-urban catchments.

The investigated river basin represents a relatively small and still poorly studied system, with only limited previous hydrogeochemical characterization available (Nisi et al., 2008), despite the strong anthropogenic pressure affecting the catchment and the strategic importance of the river for sustaining widespread agricultural activities. In this context, the study provides new insights into the response of Mediterranean temperate river systems to compound climatic and anthropogenic stressors.

The results show that prolonged drought periods favored solute accumulation, redox-sensitive transformations, and sediment-mediated release of trace elements from both natural and anthropogenic sources. These processes were subsequently disrupted during first-flush events, which represent critical hydrological thresholds controlling contaminant export and the episodic transfer of accumulated material from soils and urban surfaces into the river network. In agreement with previous studies on peri-urban catchments (Kamjunke et al., 2013; Kuhlemann et al., 2021; Smith et al., 2024), downstream urbanized sectors exhibited systematically higher TDS, nutrient, and trace-element concentrations compared to upstream rural reaches, confirming the strong influence of land use on river hydrogeochemistry under variable hydrological conditions.

A major outcome of this work is the identification of two alternating hydrogeochemical regimes: (i) a low-flow regime dominated by in-stream biogeochemical and physico-chemical processes during drought conditions, and (ii) a high-flow regime, event-driven regime characterized by rapid external inputs and enhanced hydro-mechanical mobilization during intense rainfall events. The transition between these regimes appeared particularly pronounced in catchments affected by combined climatic stress and urban pressure.

Unlike many previous river hydrogeochemical studies performed under variable discharge conditions (Godsey et al., 2009; Guo et al., 2022; Moatar et al., 2017; Saavedra et al., 2022), the present work does not simply compare concentration variations as a function of streamflow through conventional concentration–discharge (C–Q) relationships. Instead, we investigated how the relationships among geochemical species evolve during different hydrological phases, allowing a process-oriented interpretation of contaminant mobilization and biogeochemical variability during extreme events. This multiparametric geochemical approach was further supported by the analysis of stable water isotopes, which provided additional evidence for identifying hydrological stress conditions and changes in water-source contributions within the river system.

The novelty of this study, therefore, lies in the integration of event-scale geochemical and isotopic monitoring to investigate the combined effects of drought and first-flush dynamics along a rural–urban river continuum. Compared with conventional hydrochemical monitoring approaches, this coupled framework improves the interpretation of hydrological thresholds, source interactions, and short-term contaminant mobilization processes associated with drought–rewetting cycles. Although the available dataset does not allow unequivocal source apportionment of contaminants, the observed relationships among geochemical species and their temporal behavior during extreme hydrological events provide robust evidence for inferring the dominant mobilization mechanisms controlling river water quality deterioration.

Overall, the proposed approach represents a transferable framework for assessing river vulnerability under compound climatic and anthropogenic pressures and may support future monitoring strategies in similarly stressed temperate and peri-urban river systems.

## Supplementary Information

Below is the link to the electronic supplementary material.

Supplementary file1 (XLSX 29 KB)

Supplementary file2 (XLSX 10 KB)

## Data Availability

Data can be available on a request sent to the corresponding author (martina.ferrari@unifi.it).

## References

[CR2] Abernathy, M. J., Schaefer, M., Ramirez, R., Garniwan, A., Lee, I., Zaera, F., Polizzotto, M. L., & Ying, S. C. (2022). Vanadate retention by iron and manganese oxides. *ACS Earth and Space Chemistry,**6*(8), 2041–2052. 10.1021/acsearthspacechem.2c00116 36016759 10.1021/acsearthspacechem.2c00116 PMC9393891

[CR3] Adebanjo-Aina, O., & Oludoye, O. (2025). Impact of nitrogen fertiliser usage in agriculture on water quality. *Pollutants,**5*(3), 21. 10.3390/pollutants5030021

[CR101] Andrea, M., Jirka, Mark J., Carter (1975) Micro semiautomated analysis of surface and waste waters for chemical oxygen demand. *Analytical Chemistry, 47*(8), 1397–1402. 10.1021/ac60358a004 10.1021/ac60358a004 1147260

[CR4] Bianchini, G., Brombin, V., Marchina, C., & Natali, C. (2025). Isotopic evidence from the Po river under prolonged drought conditions (Northern Italy, 2022–2023). *Environments,**12*(11), 439. 10.3390/environments12110439

[CR5] Biella, R., Shyrokaya, A., Ionita, M., Vignola, R., Sutanto, S. J., Todorovic, A., Teutschbein, C., Cid, D., Llasat, M. C., Alencar, P., Matanó, A., Ridolfi, E., Moccia, B., Pechlivanidis, I., Van Loon, A., Wendt, D. E., Stenfors, E., Russo, F., Vidal, J. P., & Tallaksen, L. M. (2025). The 2022 drought needs to be a turning point for European drought risk management. *Natural Hazards and Earth System Sciences,**25*(11), 4475–4501. 10.5194/NHESS-25-4475-2025

[CR6] Bonaldo, D., Bellafiore, D., Ferrarin, C., Ferretti, R., Ricchi, A., Sangelantoni, L., & Vitelletti, M. L. (2022). The summer 2022 drought: A taste of future climate for the Po valley (Italy)? *Regional Environmental Change,**23*(1), 1. 10.1007/s10113-022-02004-z

[CR7] Bufe, A., Hovius, N., Emberson, R., et al. (2021). Co-variation of silicate, carbonate and sulfide weathering drives CO_2_ release with erosion. *Nature Geoscience,**14*, 211–216. 10.1038/s41561-021-00714-3

[CR8] Burandt, Q. C., Deising, H. B., & von Tiedemann, A. (2024). Further Limitations of synthetic fungicide use and expansion of organic agriculture in Europe will increase the environmental and health risks of chemical crop protection caused by copper-containing fungicides. *Environmental Toxicology and Chemistry,**43*(1), 19–30. 10.1002/ETC.5766 37850744 10.1002/etc.5766

[CR9] Byekwaso, F., Langergraber, G., Weigelhofer, G., Kaggwa, R., Kansiime, F., & Hein, T. (2026). Anthropogenic and natural factors impacting microbiological and physicochemical surface water quality along an urban tropical wetland. *Journal of Water, Sanitation and Hygiene for Development,**16*(2), 108–123. 10.2166/WASHDEV.2026.125

[CR10] Chelli, G. (2023). La siccità del Po nel 2022 è stata la peggiore degli ultimi due secoli. *Nature Italy*. 10.1038/D43978-023-00122-8

[CR11] Chen, D., Elhadj, A., Xu, H., Xu, X., & Qiao, Z. (2020). A study on the relationship between land use change and water quality of the Mitidja watershed in Algeria based on GIS and RS. *Sustainability,**12*(9), 3510. 10.3390/su12093510

[CR12] D.Lgs. 18/2023. (2023). Gazzetta Ufficiale. *Gazzetta Ufficiale.* https://www.gazzettaufficiale.it/eli/id/2023/03/06/23G00025/sg?utm_source=chatgpt.com.

[CR13] Diodato, N., Ljungqvist, F. C., & Bellocchi, G. (2021). Climate patterns in the world’s longest history of storm-erosivity: The Arno River Basin, Italy, 1000–2019 CE. *Frontiers in Earth Science,**9*, 637973. 10.3389/feart.2021.637973

[CR14] Fagnano, M., Agrelli, D., Pascale, A., Adamo, P., Fiorentino, N., Rocco, C., Pepe, O., & Ventorino, V. (2020). Copper accumulation in agricultural soils: Risks for the food chain and soil microbial populations. *Science of the Total Environment,**734*, 139434. 10.1016/J.SCITOTENV.2020.139434 32454337 10.1016/j.scitotenv.2020.139434

[CR15] Fuchslueger, L., Kastl, E. M., Bauer, F., Kienzl, S., Hasibeder, R., Ladreiter-Knauss, T., Schmitt, M., Bahn, M., Schloter, M., Richter, A., & Szukics, U. (2014). Effects of drought on nitrogen turnover and abundances of ammonia-oxidizers in mountain grassland. *Biogeosciences,**11*(21), 6003–6015. 10.5194/BG-11-6003-2014

[CR16] Gaillardet, J., Duprè, B., & Allegre, C. J. (1997). Chemical and physical denudation in the Amazon River Basin. *Chemical Geology*. 10.1016/s0009-2541(97)00074-0

[CR17] Gallart, F., González-Fuentes, S., & Llorens, P. (2024). Technical note: Isotopic fractionation of evaporating waters: Effect of sub-daily atmospheric variations and eventual depletion of heavy isotopes. *Hydrology and Earth System Sciences,**28*(1), 229–239. 10.5194/HESS-28-229-2024

[CR18] Gat, J. R., & Carmi, I. (1970). Evolution of the isotopic composition of atmospheric waters in the Mediterranean Sea area. *Journal of Geophysical Research,**75*(15), 3039–3048. 10.1029/JC075I015P03039

[CR19] Giustini, F., Brilli, M., & Patera, A. (2016). Mapping oxygen stable isotopes of precipitation in Italy. *Journal of Hydrology: Regional Studies,**8*, 162–181. 10.1016/J.EJRH.2016.04.001

[CR20] Godsey, S. E., Kirchner, J. W., & Clow, D. W. (2009). Concentration-discharge relationships reflect chemostatic characteristics of US catchments. *Hydrological Processes,**23*(13), 1844–1864. 10.1002/HYP.7315;PAGE:STRING:ARTICLE/CHAPTER

[CR21] Guo, D., Minaudo, C., Lintern, A., Bende-Michl, U., Liu, S., Zhang, K., & Duvert, C. (2022). Synthesizing the impacts of baseflow contribution on concentration-discharge (C-Q) relationships across Australia using a Bayesian hierarchical model. *Hydrology and Earth System Sciences,**26*(1), 1–16. 10.5194/HESS-26-1-2022

[CR22] Hammerl, V., Kastl, E. M., Schloter, M., Kublik, S., Schmidt, H., Welzl, G., Jentsch, A., Beierkuhnlein, C., & Gschwendtner, S. (2019). Influence of rewetting on microbial communities involved in nitrification and denitrification in a grassland soil after a prolonged drought period. *Scientific Reports,**9*(1), 2280. 10.1038/s41598-018-38147-5 30783152 10.1038/s41598-018-38147-5 PMC6381133

[CR23] Hariwati, L. S., Efani, A., Wardana, F. C., & Hasan, M. F. R. (2026). Impacts of land use/land cover changes on water quality in the upper brantas watershed: A spatio-temporal analysis using riparian buffer approach. *International Journal of Design & Nature and Ecodynamics,**21*(2), 299–316. 10.18280/ijdne.210201

[CR24] Hina, N. S. (2024). Global Meta-analysis of nitrate leaching vulnerability in synthetic and organic fertilizers over the past four decades. *Water,**16*(3), 457. 10.3390/w16030457

[CR25] Horve, Z., McCall, B. L., & Reisner, M. (2026). Riparian vegetation structural diversity as an indicator of riparian zone functioning in a rural to urban stream gradient. *Urban Ecosystems,**29*(1), 20. 10.1007/S11252-025-01864-7

[CR26] Kamjunke, N., Büttner, O., Jäger, C. G., Marcus, H., von Tümpling, W., Halbedel, S., Norf, H., Brauns, M., Baborowski, M., Wild, R., Borchardt, D., & Weitere, M. (2013). Biogeochemical patterns in a river network along a land use gradient. *Environmental Monitoring and Assessment,**185*(11), 9221–9236. 10.1007/S10661-013-3247-7 23780728 10.1007/s10661-013-3247-7

[CR27] Kim, K., & Lee, X. (2011). Isotopic enrichment of liquid water during evaporation from water surfaces. *Journal of Hydrology,**399*(3–4), 364–375. 10.1016/J.JHYDROL.2011.01.008

[CR28] Kittlaus, S., Milačič Ščančar, R., Kozlica, K., Weber, N., Krampe, J., Zessner, M., & Zoboli, O. (2025). Dynamics of potentially toxic elements in small rivers during high-flow events. *Journal of Contaminant Hydrology,**274*, 104659. 10.1016/J.JCONHYD.2025.104659 40618646 10.1016/j.jconhyd.2025.104659

[CR29] Krüger, M., Potthast, K., Michalzik, B., Tischer, A., Küsel, K., Deckner, F. F. K., & Herrmann, M. (2021). Drought and rewetting events enhance nitrate leaching and seepage-mediated translocation of microbes from beech forest soils. *Soil Biology and Biochemistry,**154*, 108153. 10.1016/J.SOILBIO.2021.108153

[CR30] Kuhlemann, L. M., Tetzlaff, D., & Soulsby, C. (2021). Spatio-temporal variations in stable isotopes in peri-urban catchments: A preliminary assessment of potential and challenges in assessing streamflow sources. *Journal of Hydrology,**600*, 126685. 10.1016/J.JHYDROL.2021.126685

[CR31] Kuo, Y. M., Chen, Y. H., Chiu, Y. T., & Lin, T. F. (2025). Water quality and physicochemical conditions drive chlorophylla concentrations in two connected subtropical off-stream reservoirs. *Sustainable Environment Research,**35*(1), 15. 10.1186/S42834-025-00252-2/FIGURES/5

[CR32] Leitner, S., Homyak, P. M., Blankinship, J. C., Eberwein, J., Jenerette, G. D., Zechmeister-Boltenstern, S., & Schimel, J. P. (2017). Linking NO and N_2_O emission pulses with the mobilization of mineral and organic N upon rewetting dry soils. *Soil Biology and Biochemistry,**115*, 461–466. 10.1016/J.SOILBIO.2017.09.005

[CR33] Li, S., Zhang, J., Jiang, P., & Zhang, L. (2022). Linking land use with riverine water quality: A multi-spatial scale analysis relating to various riparian strips. *Frontiers in Environmental Science*. 10.3389/fenvs.2022.1013318

[CR34] Liu, F., Conklin, M. H., & Shaw, G. D. (2024). Elevational control of isotopic composition and application in understanding hydrologic processes in the mid Merced River catchment, Sierra Nevada, California, USA. *Hydrology and Earth System Sciences,**28*(10), 2239–2258. 10.5194/HESS-28-2239-2024

[CR35] Longinelli, A., & Selmo, E. (2003). Isotopic composition of precipitation in Italy: A first overall map. *Journal of Hydrology,**270*(1–2), 75–88. 10.1016/S0022-1694(02)00281-0

[CR36] Mamun, A. A., Shams, S., & Nuruzzaman, M. (2020). Review on uncertainty of the first-flush phenomenon in diffuse pollution control. *Applied Water Science,**10*(1), 53. 10.1007/S13201-019-1127-1/FIGURES/3

[CR37] Maniquiz-Redillas, M., Robles, M. E., Cruz, G., Reyes, N. J., & Kim, L. H. (2022). First flush stormwater runoff in urban catchments: A bibliometric and comprehensive review. *Hydrology,**9*(4), 63. 10.3390/HYDROLOGY9040063

[CR38] Mikkonen, H. G., van de Graaff, R., Collins, R. N., Dasika, R., Wallis, C. J., Howard, D. L., & Reichman, S. M. (2019). Immobilisation of geogenic arsenic and vanadium in iron-rich sediments and iron stone deposits. *Science of the Total Environment,**654*, 1072–1081. 10.1016/J.SCITOTENV.2018.10.427 30841382 10.1016/j.scitotenv.2018.10.427

[CR39] Moatar, F., Abbott, B. W., Minaudo, C., Curie, F., & Pinay, G. (2017). Elemental properties, hydrology, and biology interact to shape concentration-discharge curves for carbon, nutrients, sediment, and major ions. *Water Resources Research,**53*(2), 1270–1287. 10.1002/2016WR019635

[CR40] Montanari, A., Nguyen, H., Rubinetti, S., Ceola, S., Galelli, S., Rubino, A., & Zanchettin, D. (2023). Why the 2022 Po River drought is the worst in the past two centuries. *Science Advances,**9*(32), eadg8304. 37556532 10.1126/sciadv.adg8304 PMC10411875

[CR41] Mosley, & L. M. (2015). Drought impacts on the water quality of freshwater systems; Review and integration. In *Earth-science reviews* (Vol. 140, pp. 203–214). Elsevier. 10.1016/j.earscirev.2014.11.010

[CR42] Müller, M. E., Zwiener, C., & Escher, B. I. (2021). Storm event-driven occurrence and transport of dissolved and sorbed organic micropollutants and associated effects in the Ammer River. *Southwestern Germany. Environmental Toxicology and Chemistry,**40*(1), 88–99. 10.1002/ETC.4910 33079390 10.1002/etc.4910

[CR43] Natali, S., Baneschi, I., Doveri, M., Giannecchini, R., Selmo, E., & Zanchetta, G. (2021). Meteorological and geographical control on stable isotopic signature of precipitation in a western Mediterranean area (Tuscany, Italy): Disentangling a complex signal. *Journal of Hydrology,**603*, 126944. 10.1016/J.JHYDROL.2021.126944

[CR44] Negrel, P., Allgre, C. J., Duprè, B., & Lewin, E. (1993). Erosion sources determined by inversion of major and trace element ratios and strontium isotopic ratios in river water: The Congo Basin case. In *Earth and Planetary Science Letters* (Vol. 120).

[CR45] Nisi, B., Vaselli O., Buccianti A., Minissale A., Delgado H. A., Tassi F., and Montegrossi G. (2008). Indagine geochimica ed isotopica nelle acque superficiali della Valle dell’Arno.

[CR46] Owings, S. M., Bréthous, L., Eitel, E. M., Fields, B. P., Boever, A., Beckler, J. S., Bombled, B., Lansard, B., Metzger, É., Rabouille, C., Metzger, E., & Taillefert, M. (2021). Differential manganese and iron recycling and transport in continental margin sediments of the Northern Gulf of Mexico. *Marine Chemistry,**229*, 103908. 10.1016/j.marchem.2020.103908

[CR47] Peel, H. R., Balogun, F. O., Bowers, C. A., Miller, C. T., Obeidy, C. S., Polizzotto, M. L., Tashnia, S. U., Vinson, D. S., & Duckworth, O. W. (2022). Towards understanding factors affecting arsenic, chromium, and vanadium mobility in the subsurface. *Water,**14*(22), 3687. 10.3390/W14223687 36420182 10.3390/w14223687 PMC9681123

[CR48] Ponnuchakkammal, P., Raviraj, A., Suresh Kumar, D., Kannan, B., Sumathi, S. C., Boomiraj, K., Arunadevi, K., & Vishnu, S. S. (2026). Exploring the temporal dynamics of extreme weather events in the river basin. *Theoretical and Applied Climatology,**157*(3), 146. 10.1007/S00704-026-06052-X

[CR49] Richardson, J. B. (2020). Comparing trace elements (As, Cu, Ni, Pb, and Zn) in Soils and surface waters among montane, upland watersheds and lowland, urban watersheds in New England, USA. *Water,**13*(1), 59. 10.3390/W13010059

[CR50] Ridolfi, E., Lucantonio, M., Di Baldassarre, G., et al. (2025). The interplay between the urban development of Rome (Italy) and the Tiber River floods: A review of two millennia of socio-hydrological history. *Ambio,**54*, 198–210. 10.1007/s13280-024-02078-5 39368057 10.1007/s13280-024-02078-5 PMC11662109

[CR52] SNPA. (2023). Rapporto_clima_SNPA_2022_14_07_23. Report SNPA n. 36/2023, (ISBN 978–88–448–1168–6).

[CR53] Saavedra, F. A., Musolff, A., von Freyberg, J., Merz, R., Basso, S., & Tarasova, L. (2022). Disentangling scatter in long-term concentration-discharge relationships: The role of event types. *Hydrology and Earth System Sciences,**26*(23), 6227–6245. 10.5194/HESS-26-6227-2022

[CR54] Shajib, M. T. I., Hansen, H. C. B., Liang, T., & Holm, P. E. (2019). Metals in surface specific urban runoff in Beijing. *Environmental Pollution (Barking, Essex: 1987),**248*, 584–598. 10.1016/J.ENVPOL.2019.02.039 30836240 10.1016/j.envpol.2019.02.039

[CR55] Smith, D. F., Welch, S. A., Rankin, A., Carey, A. E., & Lyons, W. B. (2024). Geochemistry of urban waters and their evolution within the urban landscape. *Frontiers in Geochemistry,**2*, 1475109. 10.3389/FGEOC.2024.1475109

[CR56] Tian, C., Wang, L., Kaseke, K. F., & Bird, B. W. (2018). Stable isotope compositions (δ^2^H, δ^18^O and δ^17^O) of rainfall and snowfall in the central United States. *Scientific Reports,**8*(1), 6712. 10.1038/s41598-018-25102-7 29712983 10.1038/s41598-018-25102-7 PMC5928101

[CR100] Venturi, S., Tassi, F., Cabassi, J., Gioli, B., Baronti, S., Vaselli, O., Caponi, C., Vagnoli, C., Picchi, G., Zaldei, A., Magi, F., Miglietta, F., Capecchiacci, F. (2020). Seasonal and diurnal variations of greenhouse gases in Florence (Italy): Inferring sources and sinks from carbon isotopic ratios. *Science of The Total Environment, 698*, 134245. 10.1016/j.scitotenv.2019.134245 10.1016/j.scitotenv.2019.134245 31494422

[CR58] Valdivielso, S., Murray, J., Custodio, E., Hassanzadeh, A., Martínez, D. E., & Vázquez-Suñé, E. (2024). Seasonal and isotopic precipitation patterns in the semi-arid and high mountain areas. *Science of the Total Environment,**925*, 171750. 10.1016/J.SCITOTENV.2024.171750 38494019 10.1016/j.scitotenv.2024.171750

[CR59] Vázquez, E., Teutscherová, N., Almorox, J., Cámara, J., Kasschau, K. D., & Benito, M. (2025). The accumulation of mineral nitrogen in soil during drying events is affected by soil management. *Soil and Tillage Research,**252*, 106623. 10.1016/J.STILL.2025.106623

[CR60] Wagner, S., Hüffer, T., Klöckner, P., Wehrhahn, M., Hofmann, T., & Reemtsma, T. (2018). Tire wear particles in the aquatic environment - A review on generation, analysis, occurrence, fate and effects. *Water Research,**139*, 83–100. 10.1016/J.WATRES.2018.03.051 29631188 10.1016/j.watres.2018.03.051

[CR61] Xiao, X., Zhan, X., Wang, Y., Liu, J., Wu, H., Liu, K., Yu, Z., & Liu, Z. (2025). The altitude effect of δ^18^O in precipitation and river water in a permafrost-underlain headwater basin, Northeastern Tibetan Plateau, China. *Journal of Hydrology,**658*, 133185. 10.1016/J.JHYDROL.2025.133185

[CR62] Yang, X., Li, T., Hua, K., & Zhang, Y. (2015). Investigation of first flushes in a small rural-agricultural catchment. *Polish Journal of Environmental Studies,**24*(1), 381–389. 10.15244/pjoes/28299

[CR63] Yang, J., Teng, Y., Song, L., & Zuo, R. (2016). Tracing sources and contamination assessments of heavy metals in road and foliar dusts in a typical mining city, China. *PLoS ONE,**11*(12), e0168528. 10.1371/JOURNAL.PONE.0168528 27992518 10.1371/journal.pone.0168528 PMC5161371

[CR64] Yang, N., & Wang, G. (2024). Spatial variation of water stable isotopes of multiple rivers in southeastern Qaidam Basin, northeast Qinghai-Tibetan Plateau: Insights into hydrologic cycle. *Journal of Hydrology,**628*, 130464. 10.1016/J.JHYDROL.2023.130464

[CR65] Zhang, X., Yu, B., Xin, Z., Cong, M., & Zhang, C. (2025). Spatial–temporal variations of river water quality under human-induced land use changes in large river basins. *Scientific Reports*. 10.1038/s41598-025-20876-z 10.1038/s41598-025-20876-z PMC12546616 41125715

[CR66] Zingraff-Hamed, A., Bonnefond, M., Bonthoux, S., Legay, N., Greulich, S., Robert, A., Rotgé, V., Serrano, J., Cao, Y., Bala, R., Vazha, A., Tharme, R. E., & Wantzen, K. M. (2021). Human–river encounter sites: Looking for harmony between humans and nature in cities. *Sustainability,**13*(5), 2864. 10.3390/su13052864

[CR67] van Vliet, M. T. H., Thorslund, J., Strokal, M., Hofstra, N., Flörke, M., Ehalt Macedo, H., Nkwasa, A., Tang, T., Kaushal, S. S., Kumar, R., van Griensven, A., Bouwman, L., & Mosley, L. M. (2023). Global river water quality under climate change and hydroclimatic extremes. *Nature Reviews Earth & Environment,**4*(10), 687–702. 10.1038/s43017-023-00472-3

[CR1] ARPAT (2023). Monitoraggio Corpi Idrici Sotterranei. Available at: https://www.arpat.toscana.it/. Accessed 2026–03–02.

[CR51] SIR – Regione Toscana (2025)—Archivio Dati. Available at: https://www.sir.toscana.it/consistenza-rete. Accessed 2026–05–22.

[CR57] UNESCO (2024). United Nations World Water Development Report. Available at: https://www.unesco.org/reports/wwdr/en/2024/s?hub=66714&utm_source=chatgpt.com. Accessed 2026–05–20.

